# Donor adipose-derived stromal cells are vasoprotectant but unable to revert acute rejection in rodent vascularized composite allotransplants

**DOI:** 10.3389/fimmu.2025.1581599

**Published:** 2025-04-28

**Authors:** Riccardo Schweizer, Pranitha Kamat, Holger J. Klein, Branislav Kollar, Matthias Waldner, Klara Stölzl, Fabienne Lehner, Souzan Salemi, Peter Bode, Daniel Eberli, Adriano Taddeo, Jan A. Plock

**Affiliations:** ^1^ Department of Plastic Surgery and Hand Surgery, University Hospital Zurich, University of Zurich, Zurich, Switzerland; ^2^ Department of Plastic Surgery and Hand Surgery, Cantonal Hospital Lucerne, University of Lucerne, Lucerne, Switzerland; ^3^ Department of Plastic Surgery and Hand Surgery, Cantonal Hospital Aarau, Aarau, Switzerland; ^4^ Department of Plastic and Hand Surgery, Medical Center - University of Freiburg, Freiburg, Germany; ^5^ Department of Urology, University Hospital Zurich, University of Zurich, Zurich, Switzerland; ^6^ Department of Pathology, University Hospital Zurich, University of Zurich, Zurich, Switzerland; ^7^ Department of Plastic Surgery and Hand Surgery, Inselspital, University of Bern, Bern, Switzerland; ^8^ Transplantation Center, University Hospital Zurich, University of Zurich, Zurich, Switzerland

**Keywords:** hand transplantation, face transplantation, immunomodulation, rejection therapy, transplant vasculitis, vasculopathy

## Abstract

**Background:**

Vascularized composite allotransplantation is successful in reconstruction of major defects of the upper extremity and face. Both rejection and vascular damage seriously endanger the outcome. The role of adipose-derived stromal cells (ASCs) in suppressing acute rejection of composite allotransplants and their short-term protective effects on vessels remains widely unexplored.

**Methods:**

Systemic and local donor-derived ASCs (CD45^−^CD29^+^CD90^+^) versus FK-506 administration was evaluated for reversal of acute rejection and vascular alterations in fully mismatched rat hind-limb transplants.

**Results:**

ASC administration upon grade II rejection significantly delayed but did not suppress progression to grade III rejection (7.6 ± 1.0 days systemic, 7.1 ± 1.1 days local vs. no cell therapy 2.9 ± 1 days; p<0.01, n=38 animals). Pro-inflammatory cytokine blood levels significantly increased in controls from grade II to grade III rejection, whereas ASC significantly lowered the levels for G-CSF, MIP-1α, MIP-3α, IL-1α, IL-1β, IL-18, and Rantes (p<0.05). Local and systemic PKH-26-labeled ASCs homed to the allograft and reversed intragraft vascular alterations in arterioles of rejecting skin and muscle, similarly to FK-506-treated controls (p<0.01).

**Conclusions:**

Although systemic and local ASC therapy reduces progression of acute rejection in vascularized composite allotransplantation, it is not able to revert rejection without additional immunosuppressive therapy. However, graft vasculitis during acute rejection is significantly reduced after cytotherapy.

## 1 Introduction

Since the inception of vascularized composite allotransplantation (VCA), over 230 procedures have been performed worldwide, offering advanced reconstructive options for anatomic and functional restoration of complex and disabling tissue defects that curtail conventional reconstructive procedures (1–3). The positive functional and quality-of-life results reported thus far need to be carefully evaluated in light of the still significant risk of allograft loss and mortality (4, 5), whereas in some cases, prosthetic solutions might be the preferred choice (6).

Acute and chronic morbidity due to lifelong systemic immunosuppression (IS), e.g., with calcineurin inhibitors, that affects kidney function promotes cancer and disturbs endocrine function (7, 8), currently limiting the indication for VCA (9, 10). Much of scientific effort is invested in finding a solution to drug-based IS, including novel modern strategies such as genetic engineering, but there is a paucity of reports that investigate alternative approaches to therapy of onset acute rejection, to reduce toxicity of corticosteroids and classical IS regimens (11).

Adipose-derived mesenchymal stem cells (ASCs) retain multiple potential therapeutic effects, including but not limited to immunomodulatory, angiogenetic, and pro-neurotropic properties (12). Our group and others have shown a beneficial effect of ASCs in peri-transplant immunomodulatory and tolerogenic regimens (13–19).

In addition to that, development of graft vasculopathy (GV) in VCA has a significant impact on the long-term outcomes of VCA and is therefore a topic of ongoing investigation. GV refers to the development of changes or abnormalities in blood vessels that supply transplanted tissue, usually in the long term due to chronic rejection, eventually leading to graft failure (20). Other than GV, very little is known about vascular alterations arising during the setting of acute rejection in VCA, whereas vasculitis has been recognized being an important issue in heart transplantation (21). We have previously shown that vascular alterations including thickening of the intima and progressive occlusion also occur during acute rejection, probably following different mechanisms than classical GV (17). Different approaches are proposed to address and prevent vascular issues in VCA, mainly based on preventive rather than therapeutic strategies taking advantage of the immunomodulatory function of immunosuppressive drugs and cell-based therapies.

While the protective effect of ASCs in terms of prevention of acute rejection and tolerance induction has been explored, to date there is no report that explores the potential of ASCs in alleviation or therapy of already manifest acute rejection in VCA, nor their potential protective effect on allograft vasculature in the setting of onset acute rejection.

Given this paucity of knowledge, we aimed at exploring the potential of local or systemically delivered donor-derived ASCs in attenuation or reversal of acute rejection in an established osteomyocutaneous rodent VCA model, compared with traditional reversal with immunosuppressive drug regimen. As secondary aim, we assessed ASC’s effect on rejection-induced vascular alterations.

## 2 Materials and methods

### 2.1 Experimental protocol

This study was performed according to Swiss animal protection law and approved by the local animal experimentation committee (Zurich, Switzerland; permission ZH 107/2015).

**Figure 1:**
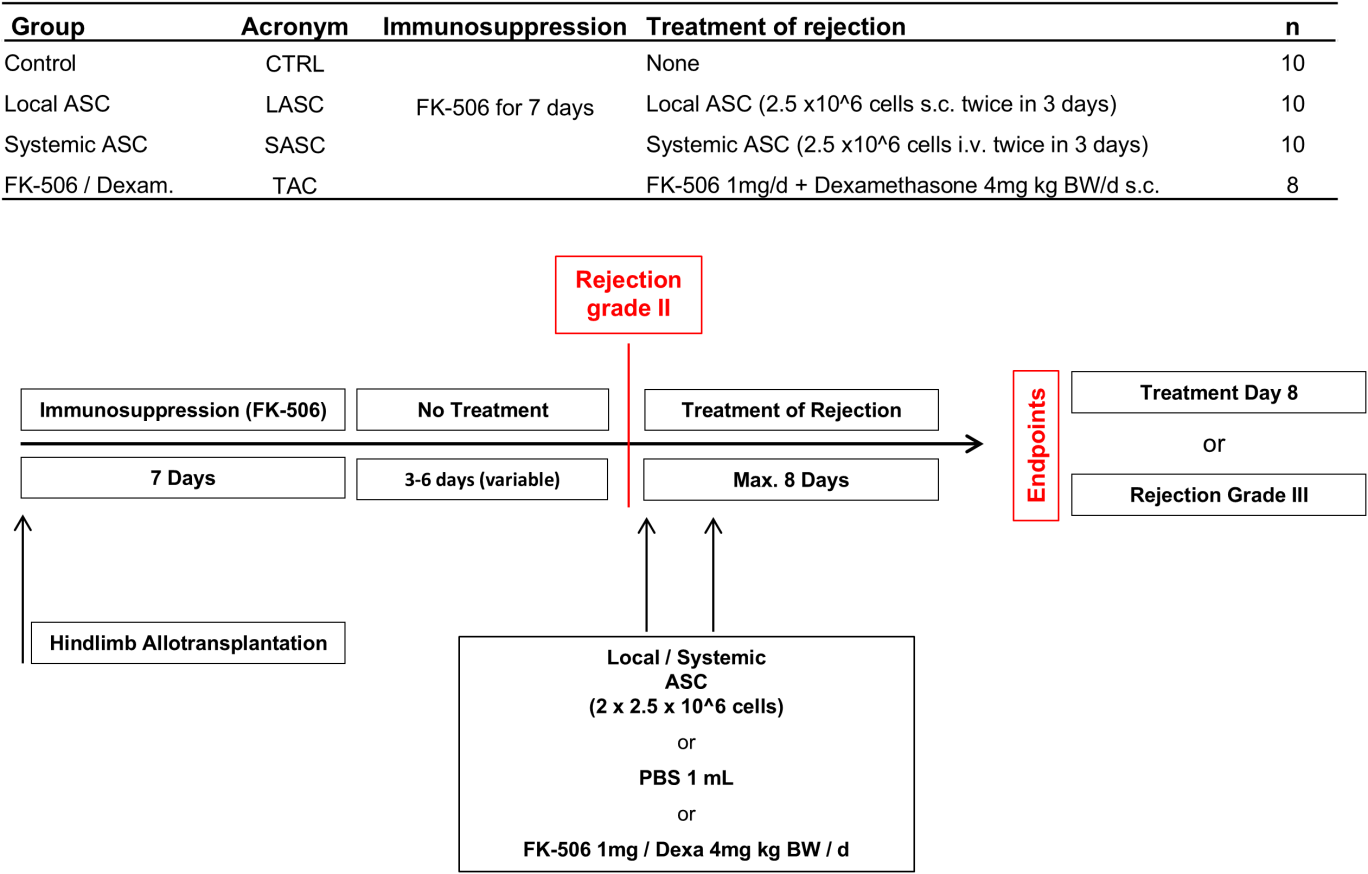
Study groups and protocol. ASC, adipose-derived stem cell.

6- to 8-week-old male Lewis (LEW, RT1 (1), recipient) and Brown Norway (BN, RT1^n^, donor) rats (Janvier or Charles River, Sulzfeld, Germany; approx. 250 g) were housed in specific pathogen-free barrier cages with food and water *ad libitum*. All recipients were treated with FK-506 (LC Labs, Woburn, MA; 0.5 mg/kg s.c.) daily from surgery to postoperative day (POD) 7 to allow wound healing and balancing of the immune system, and with dexamethasone (4 mg s.c., Galepharm AG, Switzerland) once during surgery. Four groups were formed: “CTRL” received no therapy; “LASC” received 2.5 × 10^6^ ASCs s.c. into allograft at rejection grade II and 2 days later; “SASC” received 2.5 × 10^6^ ASCs i.v. at rejection grade II and 2 days later; and “TAC” received FK-506 (0.5 mg/kg) and 4 mg dexamethasone s.c. daily (**Figure 1**). ASCs were administered in 1 mL PBS by penile vein injection (SASC) or s.c. into allograft and groin (LASC) after blood (1 mL) collection for cytokine analysis during a short isoflurane anesthesia. Before injections, skin samples were taken for baseline (BL) values. Animals were monitored daily for rejection grades (22). Rejection grade III (min. 80% of the allograft) or day 8 since start of remission therapy were defined endpoints. Clinical rejection grading occurred according to previous reports (grade 0 = normal; grade I = edema; grade II = erythema; grade III = epidermolysis) (22).

### 2.2 ASC isolation, cultivation, and characterization

To isolate ASCs, BN rat inguinal fat pads were excised and cells were isolated according to the established protocols (23). Cells were expanded *in vitro* until passage 3. For surface marker analysis, the cells were stained with anti-rat CD29, CD73, CD90, and CD45 antibodies and analyzed using an LSRII flow cytometer (BD Biosciences, San Diego, CA) with FlowJo (TreeStar Inc., Ashland, OR). For analysis of cell viability in ASCs, cells were stained with 10 μM CellTrace Calcein-AM (CellTrace Green, Life Technologies) for 30 min. at 37°C. Viable cells were detected using a fluorescence microscope.

### 2.3 Transplantation model

LEW rats received orthotopic hindlimb transplants from BN donor rats as described previously in detail (14, 17). Analgesia occurred with buprenorphine (Temgesic^®^, Eumedica Pharmaceuticals AG, Switzerland) 0.1 mg/kg BW s.c. every 6 h and paracetamol (200 mg/mL Dafalgan Sirup^®^, UPSA Switzerland AG) in the drinking water for at least for 72 h or as needed.

### 2.4 Mixed lymphocyte reaction assays

As responder cells, peripheral blood mononuclear cells (PBMCs) from the recipient were isolated as described above and stained with 5 μM carboxyfluorescein succinimidyl ester (CFSE, Thermo Fisher). Splenocytes isolated from spleens of Brown Norway were used as stimulator cells and irradiated with 30 Gy. Both responder and stimulator cells were seeded at a 1:1 ratio and cultured for 5 days in DMEM/F12 supplemented with 10% FBS, 1% Pen/Strep (Thermo Fisher), and 0.05 mM 2-mercaptoethanol (Sigma-Aldrich). After 5 days, CFSE staining was assessed on CD4^+^CD45^+^ T cells by flow cytometry and proliferation expressed with the proliferation index (PI) as determined using FlowJo software. The immunomodulatory function of ASCs was confirmed by adding donor-specific ASCs on top to abovementioned mixed lymphocyte reaction (MLR) at different concentrations in respect to responder cells (1:2, 1:1, 10:1). ASCs were cultivated as described previously (14, 17) and exosomes (EXO) isolated from the same ASCs at passages 2–4. After the seeded ASCs reached 60%–70% confluency, the culture media was changed to FBS EXO-depleted media (DMEM/F-12 [Gibco] + 10% EXO depleted FBS [Gibco] + 1% PenStrep [Gibco]) and incubated for 48 h. The media were then collected and ultrafiltered. The ultrafiltrate was separated by size exclusion chromatography and sterilized, and the EXO concentrate fraction was ultrafiltered again. The EXO that were thawed and used for the experiment, were isolated from the same ASCs, which were also used for all three MLR experiments. EXO were diluted with EXO-depleted media (1:10 [3 × 10^6^ EXO/well],1:1,10:1) and finally added to MLRs in a similar fashion as the ASCs.

### 2.5 Blood cytokine analysis

Frozen aliquots of blood plasma were thawed up over ice. For a customized multiplex immunoassay with magnetic beads, the ProcartaPlex^®^ kit (Thermo Fisher) including a set of immunological cytokines was utilized following step-by-step manufacturer’s protocol and read out with a FlexMAP 3D multiplex reader (Bio-Rad, Cressier, Switzerland). The assays were performed in duplicates following the manufacturer’s instructions. For TSG-6 an ELISA (Cusabio CSB-E17373r) was performed following step-by-step manufacturer’s protocol.

### 2.6 PKH-26 cell labeling injection

ASCs were labeled with PKH67 (Sigma Aldrich, PKH67GL-1KT) according to the manufacturer’s recommendation. A total of 1 mL PBS with cell suspension (2.5 × 10^6^ cells) was injected at multiple locations subcutaneously into the thigh and groin of the transplanted limb or systemically by tail vein punction. Samples were harvested from skin, muscle, and adipose tissue of the allograft and were embedded in Tissue-Tek (O.C.T., Thermo Fisher) and snap frozen, cryo-cut sections made and then visualized by fluorescent microscope (Leica DMi8).

### 2.7 Histopathology

Samples were harvested at endpoint from skin, fixed with 4% formalin, and paraffin-embedded, and 4-µm-thick sections were cut. The sections were stained for hematoxylin and eosin (HE) and elastin van Gieson. The slides were assessed and scored for BANFF criteria (24) and semiquantitatively for vasculopathy by a pathologist (PB) blinded to groups (25). For BANFF grade, necrosis, lymphocyte infiltration, and vasculitis scores, the pathologist assessed five representative high power fields per slide for each animal by conventional microscopy. Each high-power field was categorized according to the following scale: 0 = none/absent, 1 = minimal, 2 = moderate, 3 = extensive; and a mean score per animal created. Exclusion criteria were followed according to Cendales et al (24). Pretreatment samples of the skin were taken at grade II rejection prior to systemic or local administration of ASCs. Naive samples were retrieved from LEW rats at the time of transplantation. After fixation with formalin, samples were embedded into paraffin and stained with Van Gieson’s stain to visualize the internal elastic membrane of the arterial blood vessels. For analysis of the vessels in skin and muscle, samples with at least two identifiable vessels were included into analysis. Measurements of intima and media thickness were performed using ImageJ software (NIH, Bethesda, USA) at four different spots of the vessel wall where the tunica intima and media could be clearly identified by the internal elastic membrane. The intima/media (I/M) ratio was calculated as intima thickness (µm)/media thickness (µm) and an average calculated per vessel. All pictures were taken using a Leica DM6000 B microscope at 20–40× magnification.

For vWF immunohistochemistry, after deparaffinization and rehydration, slides were washed in Tris buffer pH 7.6 at 37°C×30 min. Antigen retrieval was performed with 0.1% pronase for 15 min at 37°C and sections blocked for 2 h with TBS+10%GS+0.05%T20 at RT. Slides were incubated with anti-vWF antibody (Abcam, Ab6994; 1:400) overnight at 4°C, followed by a secondary antibody (Abcam, Ab150077; 1:1,500) for 1.5 h at RT. After counterstaining with DAPI, slides were mounted with fluorescent mounting media (Dako).

For immunoglobulin M (IgM) immunohistochemistry, antigen retrieval was performed with sodium citrate buffer (10 mM sodium citrate, 0.05% Tween 20, pH 6.0) and blocked with TBS+5% goat serum+5%BSA+0.05% T20 for 1hr at RT. The anti-IgM antibody at 1:50 dilution (SouthernBiotech 3020-08) was applied and incubated for overnight at 4°C, followed by streptavidin Cy3 1:100 for 1 h (Sigma-Aldrich S6402).

vWF and IgM analysis was performed using ImageJ software (NIH, Bethesda, USA).

### 2.8 Statistical analysis

Prism 9.0 (GraphPad Software, La Jolla, CA) was used for statistical analysis and data presented as means ± SD, unless otherwise specified. Differences between the groups were assessed by one-way ANOVA and Bonferroni’s post-test. Graft survival was compared using Kaplan–Meier analysis and the log-rank test (Mantel–Cox) adjusted for multiple comparisons. Statistical significance is defined as * p< 0.05, ** p< 0.01, *** p< 0.001, **** p< 0.0001.

## 3 Results

### 3.1 Reduction of allo-reactivity after addition of ASCs *in vitro*

**Figure 2:**
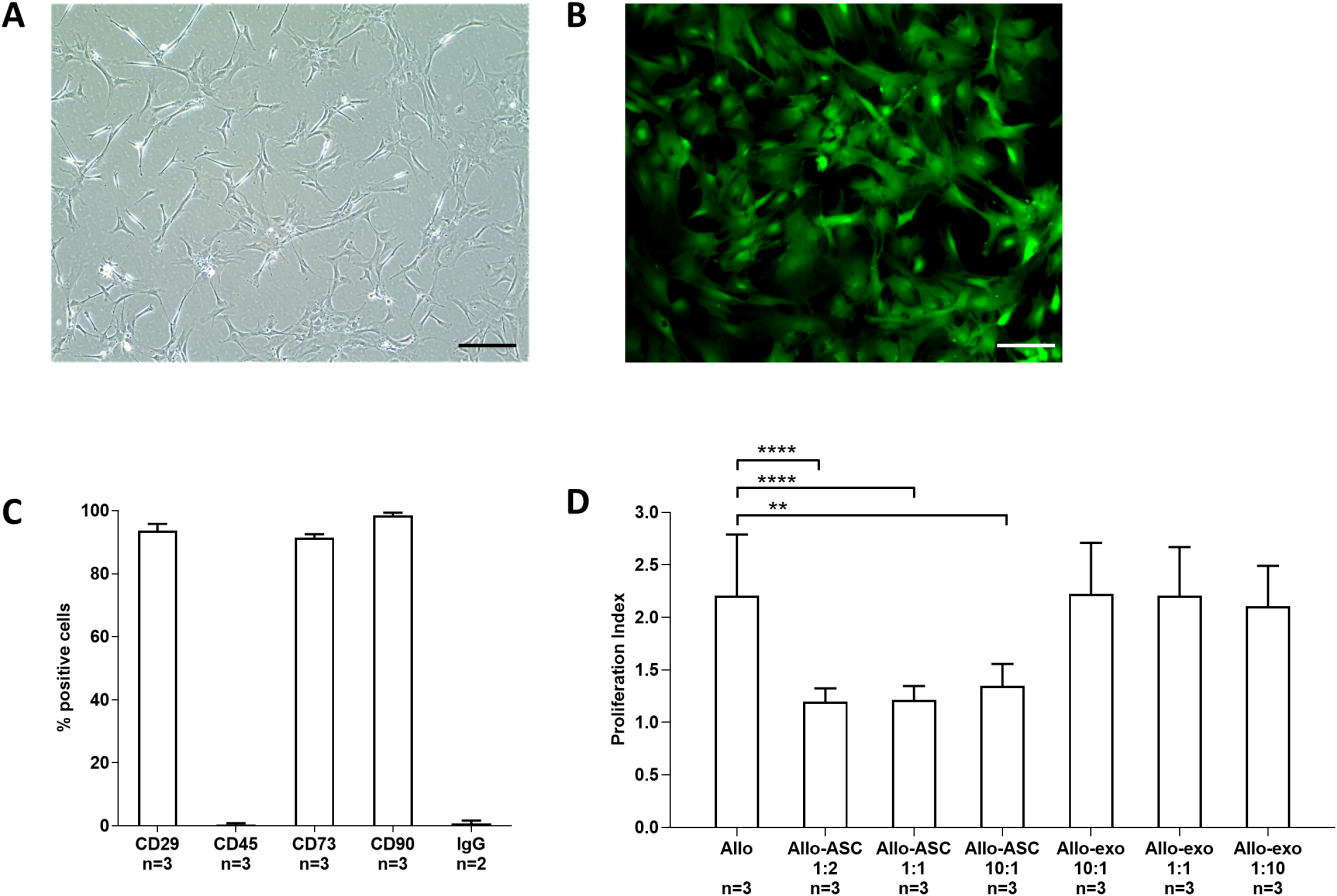
Cell characterization. Donor-specific ASCs were isolated from BN rats and culture-expanded up to passages 2–3 **(A)** and their viability assessed by Calcein AM assays **(B)**. Flow cytometry (n=3) phenotyping revealed typical marker expression (CD29, CD73, and CD90) and negativity for CD45 **(C)**. In mixed lymphocyte reaction assays (n=3), ASCs and ASC-derived EXO at different concentrations were tested for suppression of alloreaction between irradiated donor BN splenocytes and recipient PBMCs (Allo) **(D)**. Pictures taken at 10× magnification, bar = 50 µm. ASC, adipose-derived stem cell; BN, brown Norway; EXO, exosomes. ** p<0.01, **** p<0.0001.

Donor ASCs were positive for CD29 (93.70 ± 2.1%), CD73 (91.35 ± 1.2%), and CD90 (98.43 ± 0.9%) and negative for CD45 (0.32 ± 0.5%) and IgG isotype (0.75 ± 0.9%) and showed typical morphology in culture (**Figures 2A-C**; n=3). Cell viability was confirmed by Calcein AM (**Figure 2B**). ASCs, but not their EXO, significantly suppress allo-reactivity (proliferation index) of recipient T cells against BN cells in MLR assays (Allo 2.2 ± 0.58; **Figure 2D**; **Supplementary Figure 1**; n=3; Allo: ASC 1:2 1.2 ± 0.13, 1:1 1.22 ± 0.13, 10:1 1.35 ± 0.2; Allo:exo 10:1 2.22 ± 0.49 1:1 2.21 ± 0.46 1:10 2.1 ± 0.38; Allo: ASC 10:1 p<0.01, 1:1 and 1:2 p<0.0001 vs. Allo).

### 3.2 Local and systemic ASCs attenuate progression of onset rejection but are unable to achieve complete reversal as opposed to tacrolimus/dexamethasone

**Figure 3:**
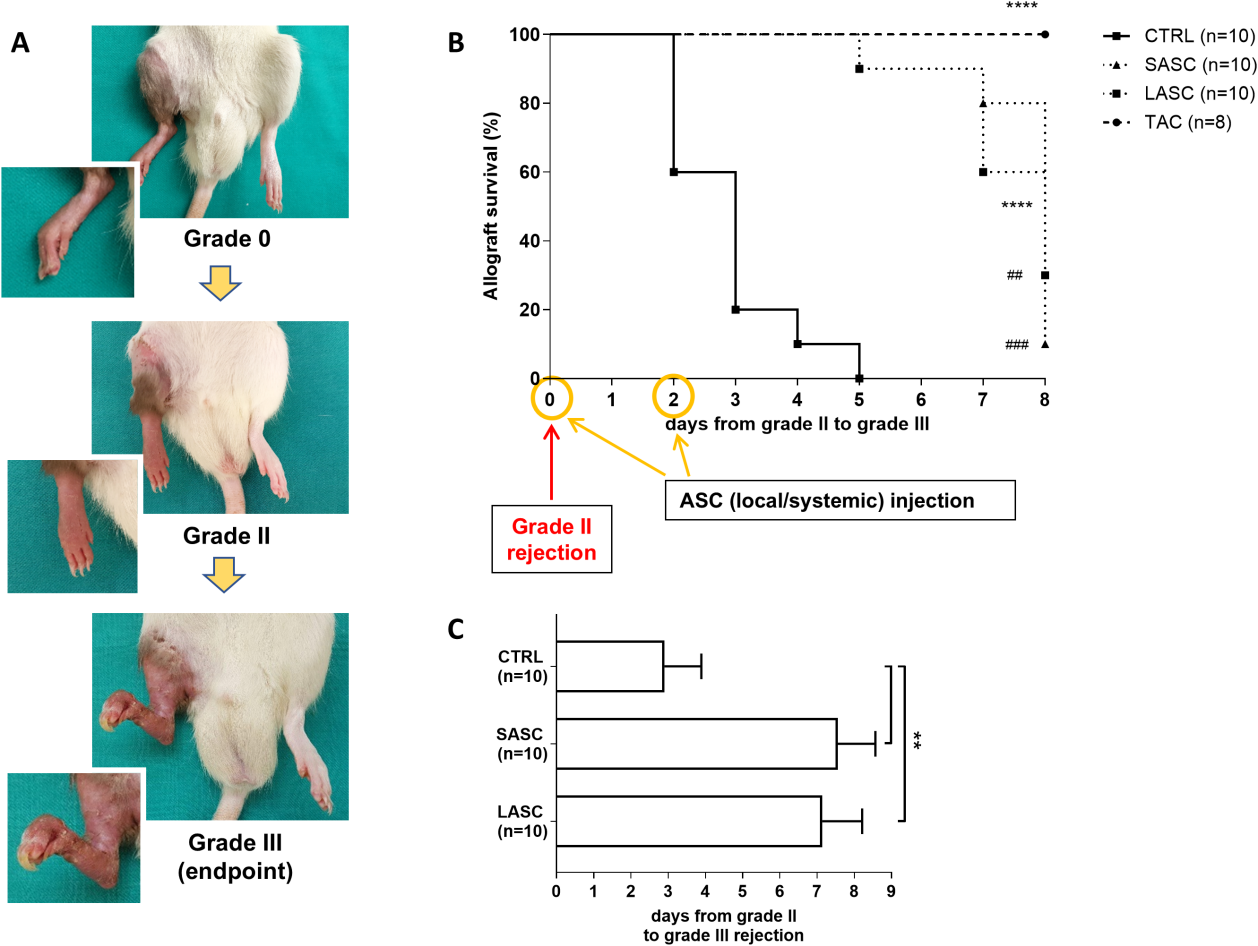
*In vivo* outcome and allograft rejection. Appearance of rejection-free interval (grade 0), to moderate rejection (grade II—timepoint for cell therapy) and severe rejection (grade III—endpoint) are depicted in picture **(A)**. Kaplan–Meier survival curves for ASC-treated groups (SASC and LASC), TAC and CTRL (****p <0.0001 vs. CTRL; ^##^ and ^###^p<0.01 and p<0.001 vs. TAC; **(B)** Numbers of elapsed days for progression from grade II to grade III rejection are depicted in graph **(C)** (**p<0.01 vs. CTRL). ASC, adipose-derived stem cell; CTRL, controls; LASC, local ASC group; SASC, systemic ASC group; TAC, FK-506-treated group.

While control animals all reached grade III rejection (**Figure 3A**) within 5 days after cessation of immunosuppression (median 3 days), in subjects receiving ASCs at grade II, the progression to grade III rejection was significantly delayed with 8 of 10 and 9 of 10, in LASC and SASC, respectively, reaching grade III rejection within day 8 of follow-up (median 8 days for both LASC and SASC; p<0.0001 vs. CTRL, p<0.01 and 0.001 vs. TAC; **Figure 3B**). Compared with that, no animal of TAC group receiving IS treatment (standard of care) progressed to grade III rejection (median undefined, p<0.0001 vs. CTRL): Seven animals out of eight reversed to grade 0, whereas one was still at grade II at endpoint.

ASCs were able to significantly postpone progression of grade II to grade III rejection in the LASC (7.1 ± 1.1 days) and SASC (7.6 ± 1 days) groups, compared with controls with no cellular therapy (2.9 ± 1.0 days; p<0.01; **Figure 3C**). The rejection timepoint and therapy follow-up for every single animal is summarized in **Supplementary Figure 2**.

### 3.3 Viable ASCs are found in the allograft after local or systemic administration and reduce pro-inflammatory cytokine blood levels during acute rejection *in vivo*

**Figure 4:**
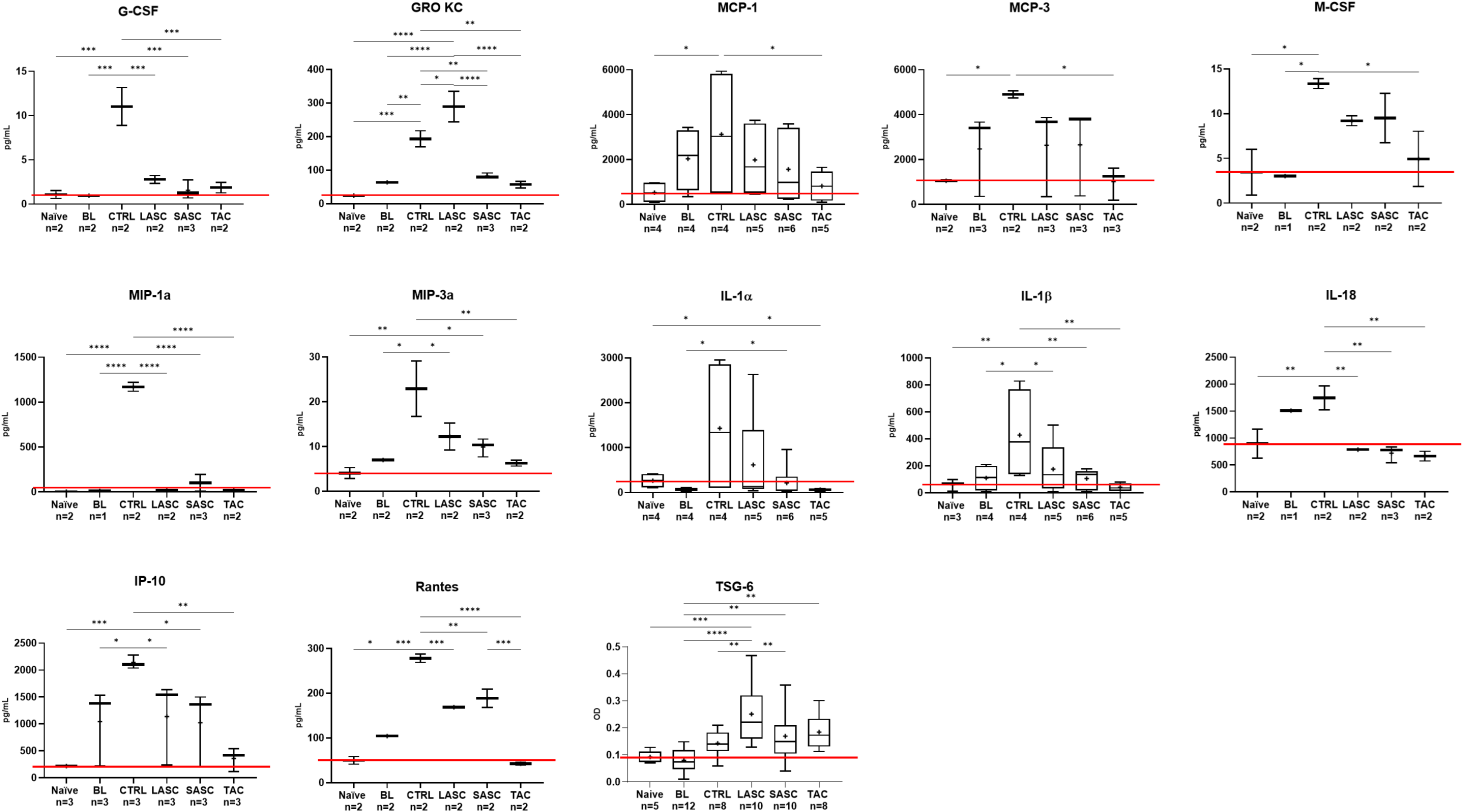
*In vivo* pro- and anti-inflammatory cytokine profile. Pro- and anti-inflammatory cytokine blood levels prior and at onset of acute rejection, and at endpoint after therapy (values in pg/mL or O.D.). *p<0.05, **p<0.01, ***p<0.001, ****p<0.0001. ASC, adipose-derived stem cell; BL, baseline = at grade II rejection, prior to therapy; CTRL, controls; O.D., optical density; LASC, local ASC group; SASC, systemic ASC group; TAC, FK-506-treated group. Red line = naïve mean value threshold.

Cytokine levels were measured in all the groups preoperatively, at grade II rejection prior to therapy and at endpoint. Blood levels of pro-inflammatory cytokines such as G-CSF (11.03 ± 3.03 pg/mL), MCP-1 (3,122 ± 299 pg/mL), MCP-3 (4,899 ± 225 pg/mL), M-CSF (13.35 ± 0.80 pg/mL), MIP-1α (1,171 ± 70 pg/mL), MIP-3α (22.91 ± 8.79 pg/mL), IL-1α (1,432 ± 1,541 pg/mL), IL-1β (428 ± 338 pg/mL), IL-2 (316 ± 140 pg/mL), IL-18 (1,746 ± 313 pg/mL), IP-10 (2,158 ± 170 pg/mL), and Rantes (278 ± 13 pg/mL) all showed an increase from grade II to grade III rejection in CTRL, compared with naïve values (all p<0.05 or lower; values in pg/mL; **Figure 4**; **Supplementary Figure 3**). Of these, G-CSF (2.784 ± 0.63 and 1.574 ± 1.05 pg/mL), MIP-1α, MIP-3α, IL-1α (616.2 ± 1127 and 213 ± 369 pg/mL), IL-1β (175.5 ± 194 and 106 ± 73 pg/mL), IL-18 (786 ± 6.5 and 720 ± 154 pg/mL), and Rantes (169 ± 2 and 189 ± 29 pg/mL) were significantly reduced in the therapeutic groups LASC and SASC, respectively, similarly to the TAC group (all p<0.05 or lower, pg/mL). GRO KC showed a similar trend in SASC and TAC (83 ± 7 and 57 ± 14.30 pg/mL) but even higher values than CTRL in LASC (193 ± 34 vs. 289 ± 64.10 pg/mL). IFN-y was significantly suppressed in SASC and TAC, compared with naïve levels (41 ± 29.6 and 40 ± 20.7 vs. 76.6 ± 17.6 pg/mL; p<0.05).

Anti-inflammatory cytokines IL-4 (LASC 25.5 ± 16.9 and SASC 28.1 ± 22.1 pg/mL) and TSG-6 (LASC 0.25 ± 0.1 and SASC 0.17 ± 0.1, O.D.) both were increased after cell therapy compared with CTRL (IL-4 19 ± 0.7 pg/mL and TSG-6 0.14 ± 0.05 O.D.; IL-4 p>0.05, TSG-6 LASC p<0.01, SASC p>0.05).

In tissue samples of the allograft at endpoint, PKH-26-labeled ASCs were found in muscle tissue of SASC animals, but not CTRL animals (as exemplary shown in **Supplementary Figure 4**). Similarly, in LASC animals, PKH-26-labeled ASCs were found in skin tissue and in the inguinal fat pad at the interface to the allograft (**Supplementary Figure 4**).

### 3.4 Pathohistological assessment and grading of allograft samples reveals no relevant improvement after cell therapy compared with controls, as opposed to complete reversal in the IS group

**Figure 5:**
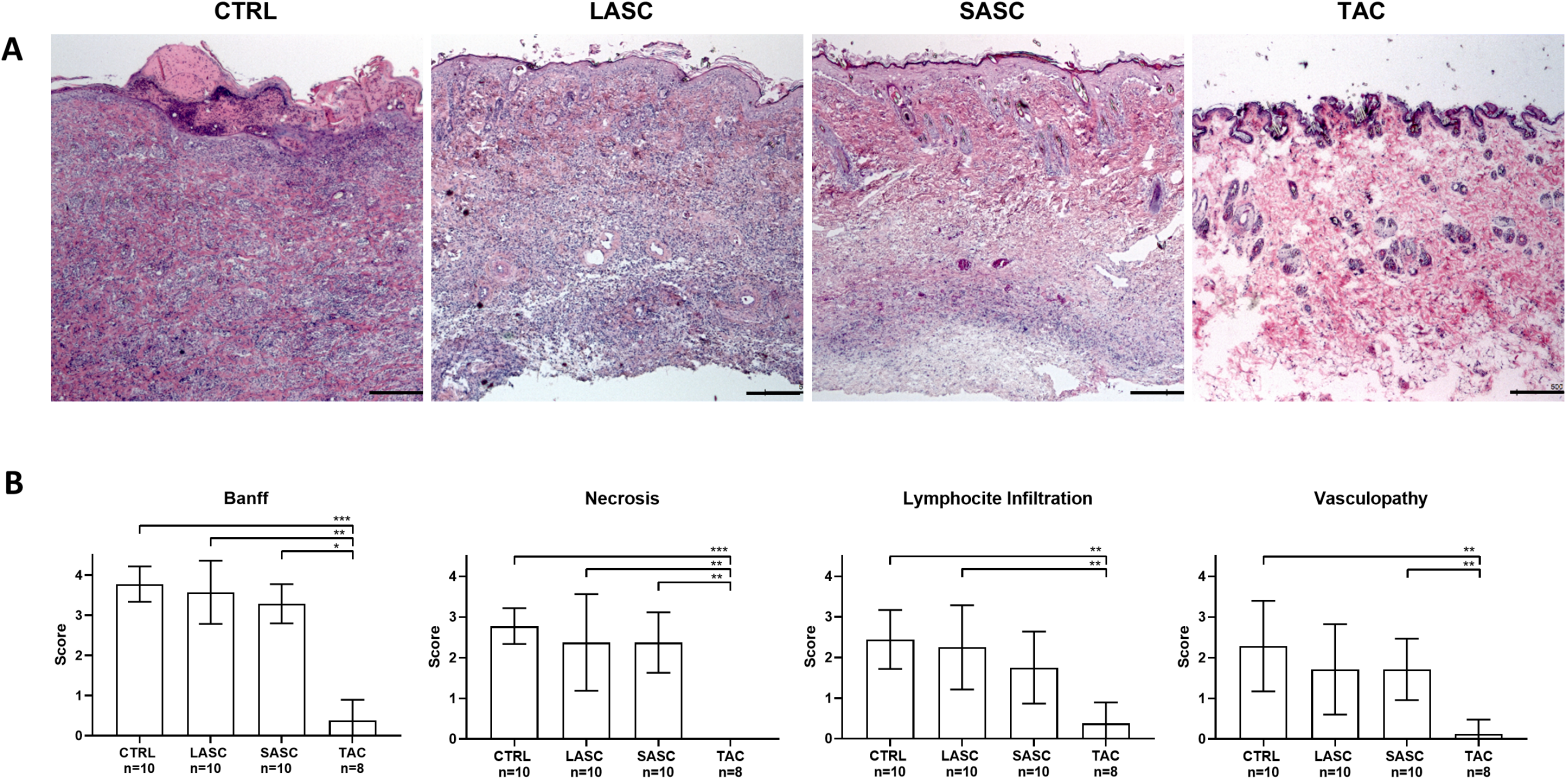
Pathohistological assessment of allograft skin samples. Exemplary pictures of HE-stained skin samples harvested from the allograft at endpoint for the different groups are provided in A. Mean scores for Banff grading, skin necrosis, infiltration, and vasculopathy are depicted in B for the different groups. Pictures taken at 5× magnification, bar = 100 µm. *p<0.05, **p<0.01, ***p<0.001. ASC, adipose-derived stem cell; CTRL, controls; LASC, local ASC group; SASC, systemic ASC group; TAC, FK-506 group.

Mean Banff scores for HE-stained skin samples (**Figure 5A**) were 3.78 ± 0.44 for CTRL, 3.57 ± 0.78 for LASC, 3.29 ± 0.49 for SASC, and 0.38 ± 0.52 for TAC (**Figure 5B**; p<0.001 vs. CTRL, p<0.01 vs. LASC, p<0.05 vs. SASC). Skin necrosis mean scores were 2.78 ± 0.44 in CTRL, 2.38 ± 1.19 in LASC, 2.38 ± 0.75 in SASC, and 0 ± 0 in TAC (p<0.01 vs. CTRL and LASC, p<0.01 vs. SASC); skin lymphocyte infiltration mean scores were 2.45 ± 0.73, 2.25 ± 1.03, and 1.75 ± 0.89 for CTRL, LASC, and SASC, respectively, compared with 0.38 ± 0.52 in TAC (p<0.01 vs. CTRL and LASC); mean scores for vasculopathy-like alterations were 2.29 ± 1.11 in CTRL, 1.71 ± 1.11 in LASC, 1.71 ± 0.76 in SASC, and 0.13 ± 0.35 in TAC (p<0.01 vs. CTRL and SASC). Pretreatment skin samples confirmed early acute rejection (histologically grades I–II) with no significant difference between the groups.

### 3.5 Local and systemic administered ASCs significantly revert and attenuate allograft vasculitis in rejecting skin and muscle arterioles, similar to the IS group

**Figure 6:**
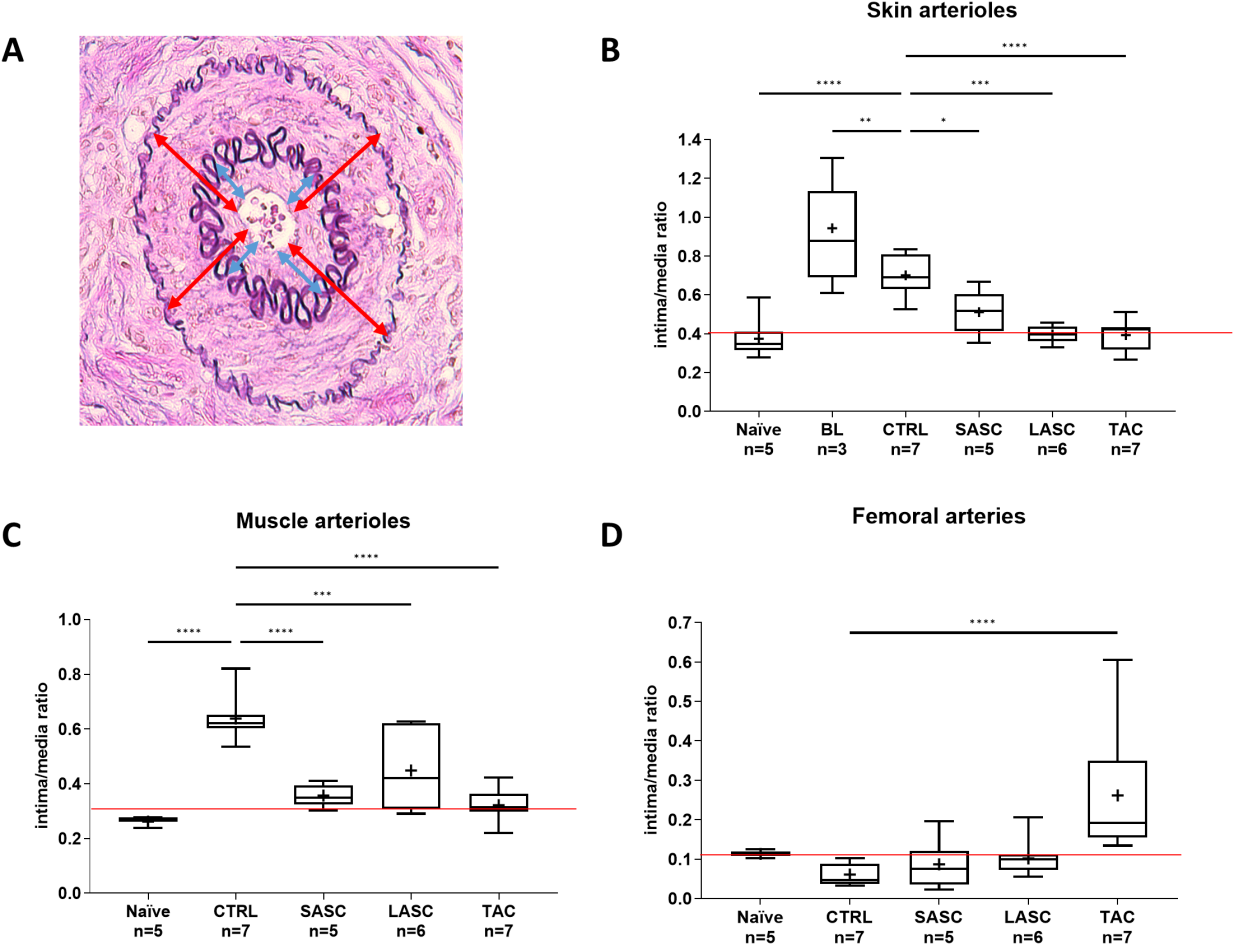
The role of ASCs in allograft vasculitis. The intima/media ratio was measured in allograft arterioles as depicted in **(A)** The intima/media ratio in skin **(B)** and muscle **(C)** arterioles, and femoral arteries **(D)** is represented for the different groups at endpoint and in naïve animals. An additional sample (BL = baseline) was taken for skin assessment prior to start of cell or FK-506 therapy. *p<0.05, **p<0.01, ***p<0.001, ****p<0.0001. ASC, adipose-derived stem cell; CTRL, controls; LASC, local ASC group; SASC, systemic ASC group; TAC, FK-506 group. Red line = naïve mean value threshold.

The I/M ratio, measured as depicted in **Figure 6A**, significantly increased in skin samples during grade II rejection (BL; 0.94 ± 0.25, p<0.0001 vs. naïve; **Figure 6B**) with respect to naïve values (0.37 ± 0.09) and slightly decreased after reaching grade III rejection in animals of the CTRL group (0.70 ± 0.11, p<0.001 vs. naïve). In animals receiving local or systemic ASCs, the I/M ratio was considerably reverted to values near naïve vessels at endpoint (SASC 0.51 ± 0.11 and LASC 0.40 ± 0.05, p>0.05 vs. naïve). The TAC group revealed similar values to naïve samples as well (0.39 ± 0.08, p>0.05 vs. naïve, LASC and SASC).

Similarly, also the I/M ratio found in arterioles of muscle samples was significantly increased in CTRL (0.64 ± 0.08, p<0.0001) compared with naïve (0.26 ± 0.02) and therapeutic groups (SASC 0.36 ± 0.04 p<0.0001, LASC 0.45 ± 0.1452 p<0.001, TAC 0.32 ± 0.06 p<0.0001; **Figure 6C**).

The I/M ratio measured in large arteries, i.e., graft femoral arteries, showed no significant difference between naïve (0.15 ± 0.015), CTRL (0.06 ± 0.02), and the cell therapy groups (SASC 0.87 ± 0.05, LASC 0.10 ± 0.04, p>0.05; **Figure 6D**). However, TAC revealed a statistically significant higher I/M ratio (0.26 ± 0.15, p<0.0001). Differentiation between arterioles smaller or larger than 40 µm revealed a similar pattern as described above, without significant differences between the two groups (showed in **Supplementary Figure 5**).

### 3.6 Local and systemic administered ASCs reduce endothelial activation (vWF) and IgM deposition in and around allograft femoral arteries

**Figure 7:**
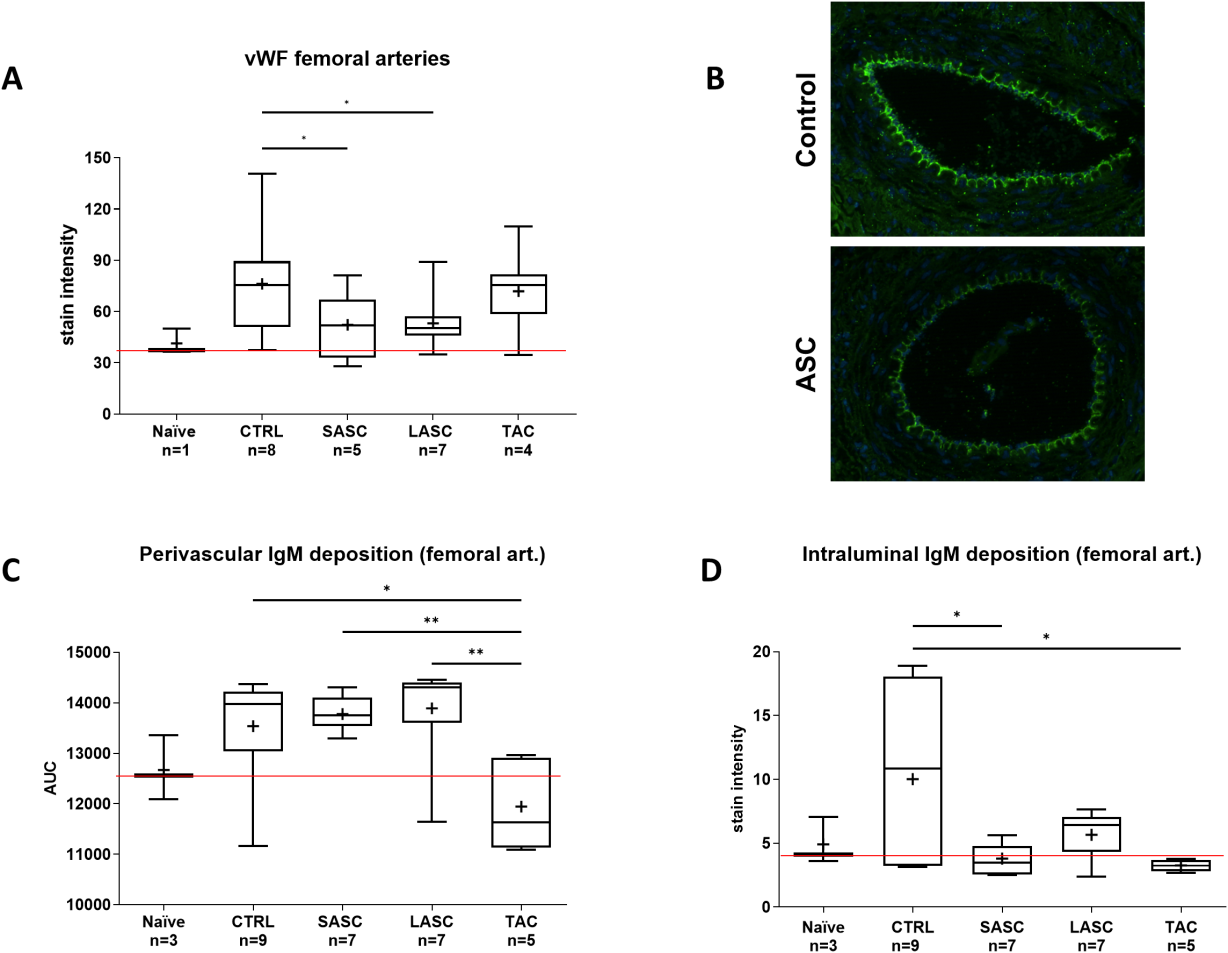
Endothelial activation in allograft greater (femoral) arteries. Endothelial activation (vWF) in graft femoral arteries are represented for each group **(A)** and exemplary fluorescence microscopy pictures provided in **(B)**. Perivascular **(C)** and intraluminal **(D)** IgM deposition in large allograft arteries is depicted for the different groups. *p<0.05, **p<0.01. ASC, adipose-derived stem cell; CTRL, controls; IgM, immunoglobulin M; LASC, local ASC group; SASC, systemic ASC group; TAC, FK-506 group; vWF, von Willebrand factor. Red line = naïve mean value threshold.

Endothelial vWF expression in femoral arteries was increased in CTRL (76.27 ± 31.11) and significantly lower in SASC and LASC (52.24 ± 18.47 and 53.12 ± 13.54, both p<0.05), whereas TAC had higher levels in a similar fashion to CTRL (71.91 ± 21.08, p>0.05; **Figures 7A, B**). Endothelial vWF expression in skin arterioles showed no significant differences between CTRL and therapeutic groups SASC, LASC, and TAC (p>0.05); however, the expression in smaller arterioles (<40µm) was approximately 10× higher than in larger ones (**Supplementary Figure 6**).

Perivascular IgM deposition was increased in CTRL (13,538 ± 1,038), SASC (13,778 ± 334), and LASC (13,892 ± 1,105) animals compared with naïve (12,670 ± 642) and, especially, TAC (11,946 ± 908) animals (p<0.05 TAC vs. CTRL, p<0.01 TAC vs. SASC and LASC; **Figure 7C**). Perivascular IgM deposition in skin arterioles showed a similar trend with CTRL significantly higher than NAÏVE and therapeutic groups (CTRL p<0.05 vs. TAC; **Supplementary Figure 7**).

Intraluminal IgM expression around femoral arteries was comparable with naïve animals (4.9 ± 1.86) and the therapeutic groups (SASC 3.8 ± 1.15, LASC 5.66 ± 1.89, and TAC 3.25 ± 0.47), whereas it was significantly higher in CTRL animals (10.02 ± 6.9, p<0.05 vs. SASC and TAC; **Figure 7D**).

## 4 Discussion

Herein, we provide first insights exploring the ability of donor-derived ASCs administered in two different modalities in mitigation of onset acute rejection in an established rodent VCA model. While both locally and intravenously injected ASCs bear potential in the setting of acute rejection in terms of modulation of rejection-related inflammation and a protective effect on allograft vasculature, the explored cell therapy protocol disclosed its limits when it comes to suppression and reversal of acute rejection and allograft survival in its actual form.

Indeed, ASC therapy alone was able to postpone, but not to suppress progression of rejection: while most control animals reached rejection grade III within 2–3 days from diagnosis of grade II rejection, animals treated with ASCs reached grade III significantly later (7–8 days), and few animals still at grade II rejection at follow-up day 8 (chosen as endpoint with the intention to assess tissue and blood samples in the acute phase). The present dosing and repetitive ASC administration were chosen according to previous own and other reports (17, 26), where we could show that repeated cell administration brings further benefit compared with a single dose. The total cell dosage in our protocol was determined in accordance with other reports (14, 17, 27, 28). We did not test different doses in this study; however, this therapeutic scheme could be adapted to daily injections and/or higher cell doses with potential increased benefit, as shown in preventive and tolerogenic strategies in VCA (17) or in cardiac allotransplantation (29). In the clinical setting, one would start earlier with cell therapy (during rejection grades I–II) and possibly supported by a low-dose drug IS regimen. We started cellular therapy at rejection grade II to challenge cell therapy and better discriminate between the groups; additionally, grade II rejection is easier to diagnose, since in this VCA model, grade I rejection can occur with only mild swelling, possibly overlapping with postoperative swelling. One limitation is that we did not assess histological grading prior to initiation of therapy, and we assessed pretreatment skin samples only retrospectively, although confirming grade I–II rejection with no significant intergroup difference.

Under the line, ASCs alone seem insufficient for complete reversal of rejection without the addition of drug-based IS, regardless whether delivered locally or systemically. However, Vandermeulen et al. found no synergistic effect applying bone marrow-derived MSCs in a rat model of liver transplantation, when compared with Everolimus alone (30). Casiraghi et al. found in a murine kidney transplant model that timing of syngeneic MSC infusion is crucial, given that administration of MSCs and complement antagonists during transplantation was able to induce regulatory T cells (T regs) and MSC homing to lymphatic tissues, but infusion 2 days later did not (31). Direct autologous ASC administration through portal infusion at the time of transplantation without standard IS has been found beneficial in rodent liver transplantation by T reg induction and cytokine regulation, reducing acute rejection and significantly prolonging graft survival (32). Similar findings were published by Watanabe et al. in rat lung transplantation, underscoring the synergistic effects of ASCs and tacrolimus, compared with ASCs alone (33). They, however, did not investigate how much IS could be reduced with the aid of cell therapy, keeping a similar positive effect on control of rejection.

In human liver transplantation, MSCs (1 × 10 (6) cells/kg) administered i.v. were able to reduce acute rejection-related serum markers and histopathological rejection findings (34) through upregulation of T regs and increasing the T reg/T helper ratio. On the other hand, another clinical report showed the contrary in liver transplantation (35). This discrepancy between the studies is probably also due to different isolation, culture, and administration methods of cell therapies and different antigenicity of the organs. MSC therapy could show synergistic effects with other cell types, such as T regs, as reported in the setting of acute brain injury (36), but there is no report exploring this in transplantation or VCA thus far.

Noteworthily, more recent reports highlight different potential cell therapies that go beyond the use of MSCs/ASCs, such as T regs, macrophage regulatory cells, or dendritic cells (26). T regs are a very promising cell type that could help in transplant rejection due to their immunomodulatory function and might be able to promote allograft tolerance, although it is unclear if they would be helpful in the hostile milieu during manifest acute rejection (37). There is some evidence that T regs can synergistically improve MSCs’ immunomodulatory function; thus, future research should perhaps focus on cellular therapies that include different kinds of cells to improve their efficacy in mediating or mitigating acute rejection (37), possibly in combination with immunotherapies (e.g., ustekinumab and secukinumab (38)). Based on previous animal experiments, a group investigated the safety and utility of expanded autologous T regs in mitigation or control of early transplant inflammation in kidney transplant recipients, with promising results and no adverse events (39). Similarly, in a pilot study, Todo et al. found that administration of *ex vivo* expanded T regs promoted weaning of liver transplant patients from immunosuppression in a stable manner in 70% of patients (40 *). A* phase I/IIa clinical trial reported a significant beneficial effect of T reg administration and success in reduction from triple- or double-IS therapy to monotherapy in 73% of kidney transplant patients by reduction of T-cell activation by T regs (41). Reestablishment of immune cell homeostasis after T reg therapy was effective and safe, allowing stable monotherapy, with no increased episodes of acute rejection and less infectious sequelae than under traditional IS (42). Macrophage regulatory cells are another set of cells capable of dampening allogeneic rejection responses and potentially allowing minimization of conventional IS therapy. They have been used safely in a pilot study in human kidney transplantation, reducing maintenance IS in most patients; however, this was not explored in the setting of acute rejection (43). Clearly, antigenicity of skin and mucosa containing allografts is thought to be much more challenging than SOT such as liver and kidney, and as stated before, most reports explored the use of cell therapies for weaning of IS but not at the time of ongoing acute rejection.

The advantage of using donor- over recipient-derived cells is not yet elucidated from an immunological perspective; from a clinical point of view, the advantage is that a large quantity of ASCs can be retrieved from brain-dead donors at the time of VCA harvest and expanded or even frozen in high aliquots for later use, the reason why we opted for this source and timing. Donor-derived ASCs were able to reduce the alloresponse in MLRs at different concentrations after their expansion, as previously shown (14, 17, 44, 45); however, sole ASC-derived EXO were not able to suppress the alloresponse in our hands (46).

Local use of ASCs in rat VCAs has been found more effective than systemic administration as a tolerance-inducing immunomodulatory regimen (47). The present study first shows that ASCs are capable of exerting beneficial therapeutic effects even when administered locally into hostile microenvironments such as grade II rejection, which is characterized by moderate but significant cellular infiltrates and tissue inflammation. This state of inflammation, on the other hand, could promote anti-inflammatory properties of MSCs and/or provide guidance for homing of the cells when administered systemically through local secretion of chemoattracting factors (48). For example, the immunomodulatory function of MSC is activated by IFN-y (49); thus, IFN-y-conditioned MSCs could be a promising approach (50). Enhancing cell durability and resilience in inflamed tissue may be achieved by applying hypoxic or alternative conditioning to ASCs during culture, expansion, and pre-administration, as suggested previously (51–53). We expected increased efficacy of locally injected ASCs compared with systemic administration, supposing that they would engraft and exert their beneficial effect “on site”: however, our data did not show any significant difference between the two route of administration.

Intracellular PKH-26-labeled cells were found in the graft at endpoint, suggesting that at least part of the cells injected intravenously homed and engrafted at the site of need. To avoid excessive entrapment in filtering organs and life-threatening lung embolies, intravenous injections occurred very slowly (54). As a limitation, this is only a descriptive finding and does not assess the quantity of cells that were injected intravenously and the percentage that was found in the graft, out of scope in the present study. After local injection, cells were found in both fat pad and allograft skin several days after injection: other reports have shown that topically applied cells remain at the site of injection and as identified by bioluminescence technology (55, 56).

Blood cytokine analysis revealed intriguing results: pro-inflammatory cytokines significantly increased in controls without therapy (G-CSF, IL-1α, IL-1β, Rantes, IP-10, IL-18), as it would be expected during acute rejection. However, in animals receiving immunomodulating ASC therapy, a significant set of pro-inflammatory cytokines was reduced and had values toward those under drug-based IS therapy (TAC). This highlights and confirms ASCs’ capacity to regulate the immune system even during acute rejection, although in the present study this was not sufficient for clinical reversal of rejection. IL-18, which promotes transplant rejection, was significantly reduced in ASC groups (57, 58): indeed, IL-18 upregulates IP-10 (CXCL10) and promotes recruitment of activated T cells (Th1), which is in line with increased IP-10 levels mostly in the control group as found in our study (59). IL-18 induces Rantes (CCL5), which in turn promotes recruitment of Th1 T cells, and was clearly decreased in animals receiving ASCs compared with controls. Similarly, Friedman et al. reported values for Rantes, MIP-1α, MIP-3α, and chemokine IP-10 that align with our findings (58). MCP-1, MCP-3, and IP-10 were significantly increased at baseline (grade II), confirming our previous findings and aligning with other authors’ observations, identifying them as early rejection markers (19, 58, 59): these were significantly reduced after ASC treatment. IFN-y was found decreased in graft samples compared with naïve skin also by Brandacher’s group (60).

Interestingly, blood IL-4 and TSG-6 levels, known having anti-inflammatory properties, were higher in ASC groups compared with CTRL, underscoring their immunomodulating action (61, 62). IL-4, produced by Th2 cells, regulates immune responses by regulatory T cells and is primarily anti-inflammatory: it was not increasingly upregulated compared with naïve values, but blood levels were higher than at grade II rejection and in controls (63). TSG-6, an anti-inflammatory cytokine upregulated upon TNF-α secretion, was significantly increased in the LASC group, comparable with levels in the TAC group. This correlated with a better outcome, with three animals at grade II at the endpoint, compared with just one in the systemic group (61). Strikingly, IL-10 was not increased in the ASC groups compared with controls, consistent with another study (64). This could be due to the use of culture-expanded ASCs rather than the stromal vascular fraction. Inhibition of IL-10 production is a critical factor in the ability of FK-506 to reverse ongoing allograft rejection (65). It is important to note that the effects of these cytokines are complex and context-dependent, and they can act in different ways in different biological systems.

Pathohistological analysis of tissues samples according to Banff criteria (24) histologically confirmed the clinical gradings for all groups, showing cellular rejection for all animals other than the TAC group, despite the ASC groups having slightly, not significant, lower scores. Signs for vasculitis-like alterations in skin samples as assessed by our group-blinded pathologist were identified in CTRL, LASC, and SASC, with no relevant difference between the groups, but not in TAC (25).

We further assessed arterioles of skin samples by Elastin-specific staining for vascular alterations (e.g., intimal thickening) quantitatively, as opposed to the qualitative and semiquantitative assessment by our pathologist (17). The I/M ratio, as expression of intimal proliferation, significantly increased in skin arterioles of grade II rejecting animals, whereas in grade III rejecting animals, intima thickness decreased again in CTRL animals. One putative reason might be the acutely inflamed intimal layer being thicker (i.e., “endothelialitis” or “intimal arteritis”) than an already deteriorating intima during rejection grade III (25). ASC-treated animals revealed significant lower I/M ratios in skin and muscle arterioles, similar to vessels in the drug-based reversal group (TAC) and naïve animals, highlighting and confirming the positive effect of ASCs on “graft vasculitis” during acute rejection (17). vWF expression as a marker for endothelial activation did not correlate with the pattern found for intimal thickening in arterioles, suggesting that other mechanisms might be involved. However, we noticed increased intravascular IgM deposition in arterioles of CTRL animals, suggesting vascular damage due to antibody-mediated rejection, which was reduced in the cell therapy and TAC groups. We acknowledge the limitation that we did not additionally assess IgG deposition in this study, which would potentially further confirm the antibody-mediated mechanism of vascular deterioration.

We found a different picture in femoral arteries: while there were no significant differences between the therapeutic groups and control animals, FK-506-treated animals had significantly higher I/M ratios, accompanied by higher vWF expression in these vessels. Tacrolimus-associated thrombotic microangiopathy has been described as rare but severe complication in the past (66, 67), but the mechanism in our setting remains as interesting as unclear. In clinical pediatric heart transplantation, increased vWF levels correlated well with GV (68). In addition, also in large arteries, IgM deposition was significantly higher in CTRL animals compared with other groups. The different degrees of endothelial activation and intimal thickening in the different branches and levels of the vascular three are something novel and never been shown for VCA and could be part of the “split rejection” phenomenon, as described by Pomahac (69). Tissue and systemic cytokine and chemokine profiles could play an important role in early diagnosis of GV (70) and show distinct patterns in GV. Disruption of anticoagulant pathways and fibrin deposition is predictive for development of early GV (71), and there are many cytokines that are upregulated during rejection and are suspected of promoting GV, such as MCP1- and 3 (72, 73), RANTES, and IP-10 (74, 75). IL-1β and 18 are involved in complement-endothelial activation (76, 77). All these factors were significantly upregulated in CTRL and reduced in the ASC groups and correlated with increased intimal hyperplasia in graft skin and muscle arterioles of controls, so this could hold true also in the setting of acute, T-cell-mediated rejection. In fact, we would like to point out that GV usually is a term associated with vascular alteration and deterioration in the setting of chronic, antibody-mediated rejection: in the short-term setting of this study, we believe that graft vasculitis (or “endothelialitis”) is a better term to describe the vascular alterations found during acute rejection and needs to be distinguished from “graft vasculopathy” (25). Our results are in line with recent emerging evidence that acute T cell-mediated rejection can be associated with lymphocytic vasculitis, as described also in the consensus paper of the 15th Banff Conference with regard to heart transplantation, which could be correlated with intimal thickening, as observed in our study (21, 78). In fact, endothelial cells hold a key role in acute rejection, can act as antigen-presenting cells, and are involved in both cell- and antibody-mediated rejection. They are the first location where the complex interplay between donor and recipient cells takes place; before the immune cells, merely CD8 effector T cells progressively infiltrate the allograft (38).

In conclusion, despite some promising findings in terms of cytokine regulation and vascular protection, the use of ASCs as stand-alone treatment of acute rejection is insufficient as of now without aid of classic IS. The benefit of different timepoints, higher dosage, combination of local and systemic delivery, or combination with other cell types, e.g., T regs, should be further investigated.

## Data Availability

The raw data supporting the conclusions of this article will be made available by the authors, without undue reservation.

## References

[1] Shores JT Brandacher G Lee WP. Hand and upper extremity transplantation: an update of outcomes in the worldwide experience. Plast Reconstr Surg. (2015) 135:351e–60e. doi: 10.1097/prs.0000000000000892 25401735

[2] Siemionow M. The decade of face transplant outcomes. J materials science Materials Med. (2017) 28:64. doi: 10.1007/s10856-017-5873-z 28303433

[3] Kollar B Tasigiorgos S Dorante MI Carty MJ Talbot SG Pomahac B. Innovations in reconstructive microsurgery: Reconstructive transplantation. J Surg Oncol. (2018) 118:800–6. doi: 10.1002/jso.25147 30098294

[4] Longo B Pomahac B Giacalone M Cardillo M Cervelli V. 18 years of face transplantation: Adverse outcomes and challenges. J plastic reconstructive aesthetic surgery: JPRAS. (2023) 87:187–99. doi: 10.1016/j.bjps.2023.09.043 37879143

[5] Glahn JZ Huelsboemer L Pomahac B. Face transplantation, social death, and bias in health care resource allocation. Ann Surg. (2024) 279:920–2. doi: 10.1097/SLA.0000000000006195 38214163

[6] Noel OF Dumbrava MG Daoud D Kammien AJ Kauke-Navarro M Pomahac B. Vascularized composite allograft versus prosthetic for reconstruction after facial and hand trauma: comparing cost, complications, and long-term outcome. Ann Plast Surg. (2024) 92:100–5. doi: 10.1097/SAP.0000000000003731 37962243

[7] Ravindra KV Wu S McKinney M Xu H Ildstad ST. Composite tissue allotransplantation: current challenges. Transplant Proc. (2009) 41:3519–28. doi: 10.1016/j.transproceed.2009.08.052 19917338

[8] Doycheva I Amer S Watt KD. *De novo Mali*gnancies after transplantation: risk and surveillance strategies. Med Clinics North America. (2016) 100:551–67. doi: 10.1016/j.mcna.2016.01.006 27095645

[9] Gorantla VS Brandacher G Schneeberger S Zheng XX Donnenberg AD Losee JE. Favoring the risk-benefit balance for upper extremity transplantation--the Pittsburgh Protocol. Hand Clinics. (2011) 27(4):511–20, ix-x. doi: 10.1016/j.hcl.2011.08.008 22051391

[10] Schneeberger S Gorantla VS Brandacher G Zeevi A Demetris AJ Lunz JG. Upper-extremity transplantation using a cell-based protocol to minimize immunosuppression. Ann Surg. (2013) 257:345–51. doi: 10.1097/SLA.0b013e31826d90bb PMC4162482 23001085

[11] Kauke-Navarro M Noel OF Knoedler L Knoedler S Panayi AC Stoegner VA. Novel strategies in transplantation: genetic engineering and vascularized composite allotransplantation. J Surg Res. (2023) 291:176–86. doi: 10.1016/j.jss.2023.04.028 37429217

[12] Parekkadan B Milwid JM. Mesenchymal stem cells as therapeutics. Annu Rev Biomed Eng. (2010) 12:87–117. doi: 10.1146/annurev-bioeng-070909-105309 20415588 PMC3759519

[13] Kuo YR Chen CC Goto S Lin PY Wei FC Chen CL. Mesenchymal stem cells as immunomodulators in a vascularized composite allotransplantation. Clin Dev Immunol. (2012) 2012:854846. doi: 10.1155/2012/854846 23227090 PMC3514826

[14] Plock JA Schnider JT Zhang W Schweizer R Tsuji W Kostereva N. Adipose- and bone marrow-derived mesenchymal stem cells prolong graft survival in vascularized composite allotransplantation. Transplantation. (2015) 99:1765–73. doi: 10.1097/TP.0000000000000731 26102613

[15] Kuo YR Chen CC Goto S Huang YT Wang CT Tsai CC. Immunomodulatory effects of bone marrow-derived mesenchymal stem cells in a swine hemi-facial allotransplantation model. PloS One. (2012) 7:e35459. doi: 10.1371/journal.pone.0035459 22558153 PMC3338845

[16] Kuo YR Chen CC Goto S Lee IT Huang CW Tsai CC. Modulation of immune response and T-cell regulation by donor adipose-derived stem cells in a rodent hind-limb allotransplant model. Plast Reconstr Surg. (2011) 128:661e–72e. doi: 10.1097/PRS.0b013e318230c60b 22094768

[17] Plock JA Schnider JT Schweizer R Zhang W Tsuji W Waldner M. The influence of timing and frequency of adipose-derived mesenchymal stem cell therapy on immunomodulation outcomes after vascularized composite allotransplantation. Transplantation. (2017) 101:e1–e11. doi: 10.1097/tp.0000000000001498 27893612

[18] Pan H Zhao K Wang L Zheng Y Zhang G Mai H. Mesenchymal stem cells enhance the induction of mixed chimerism and tolerance to rat hind-limb allografts after bone marrow transplantation. J Surg Res. (2010) 160:315–24. doi: 10.1016/j.jss.2008.09.027 19524257

[19] Schweizer R Taddeo A Waldner M Klein HJ Fuchs N Kamat P. Adipose-derived stromal cell therapy combined with a short course nonmyeloablative conditioning promotes long-term graft tolerance in vascularized composite allotransplantation. Am J Transplant. (2020) 20:1272–84. doi: 10.1111/ajt.15726 31774619

[20] Kollar B Kamat P Klein HJ Waldner M Schweizer R Plock JA. The significance of vascular alterations in acute and chronic rejection for vascularized composite allotransplantation. J Vasc Res. (2019) 56:163–80. doi: 10.1159/000500958 31266018

[21] Duong Van Huyen JP Fedrigo M Fishbein GA Leone O Neil D Marboe C. The XVth Banff Conference on Allograft Pathology the Banff Workshop Heart Report: Improving the diagnostic yield from endomyocardial biopsies and Quilty effect revisited. Am J Transplant. (2020) 20:3308–18. doi: 10.1111/ajt.16083 32476272

[22] Sacks JM Kuo YR Taieb A Breitinger J Nguyen VT Thomson AW. Prolongation of composite tissue allograft survival by immature recipient dendritic cells pulsed with donor antigen and transient low-dose immunosuppression. Plast Reconstr Surg. (2008) 121:37–49. doi: 10.1097/01.prs.0000293754.55706.7f 18176204

[23] Bunnell BA Flaat M Gagliardi C Patel B Ripoll C. Adipose-derived stem cells: isolation, expansion and differentiation. Methods. (2008) 45:115–20. doi: 10.1016/j.ymeth.2008.03.006 PMC3668445 18593609

[24] Cendales LC Kanitakis J Schneeberger S Burns C Ruiz P Landin L. The Banff 2007 working classification of skin-containing composite tissue allograft pathology. Am J Transplant. (2008) 8:1396–400. doi: 10.1111/j.1600-6143.2008.02243.x 18444912

[25] Dzhonova DV Olariu R Leckenby J Banz Y Prost JC Dhayani A. Local injections of tacrolimus-loaded hydrogel reduce systemic immunosuppression-related toxicity in vascularized composite allotransplantation. Transplantation. (2018) 102:1684–94. doi: 10.1097/tp.0000000000002283 29794937

[26] Kauke M Safi AF Panayi AC Palmer WJ Haug V Kollar B. A systematic review of immunomodulatory strategies used in skin-containing preclinical vascularized composite allotransplant models. J plastic reconstructive aesthetic surgery: JPRAS. (2022) 75:586–604. doi: 10.1016/j.bjps.2021.11.003 34895853

[27] Davis TA Anam K Lazdun Y Gimble JM Elster EA. Adipose-derived stromal cells promote allograft tolerance induction. Stem Cells Trans Med. (2014) 3:1444–50. doi: 10.5966/sctm.2014-0131 PMC4250215 25411475

[28] Solari MG Srinivasan S Boumaza I Unadkat J Harb G Garcia-Ocana A. Marginal mass islet transplantation with autologous mesenchymal stem cells promotes long-term islet allograft survival and sustained normoglycemia. J Autoimmun. (2009) 32:116–24. doi: 10.1016/j.jaut.2009.01.003 19217258

[29] Wang F Chen X Li J Wang D Huang H Li X. Dose- and time-dependent effects of human mesenchymal stromal cell infusion on cardiac allograft rejection in mice. Stem Cells Dev. (2021) 30:203–13. doi: 10.1089/scd.2019.0300 33371825

[30] Vandermeulen M Erpicum P Bletard N Poma L Jouret F Detry O. Effect of the combination of everolimus and mesenchymal stromal cells on regulatory T cells levels and in a liver transplant rejection model in rats. Front Immunol. (2022) 13:877953. doi: 10.3389/fimmu.2022.877953 35757737 PMC9226583

[31] Casiraghi F Todeschini M Azzollini N Cravedi P Cassis P Solini S. Effect of timing and complement receptor antagonism on intragraft recruitment and protolerogenic effects of mesenchymal stromal cells in murine kidney transplantation. Transplantation. (2019) 103:1121–30. doi: 10.1097/TP.0000000000002611 PMC6934941 30801518

[32] Gao W Zhang L Zhang Y Sun C Chen X Wang Y. Adipose-derived mesenchymal stem cells promote liver regeneration and suppress rejection in small-for-size liver allograft. Transpl Immunol. (2017) 45:1–7. doi: 10.1016/j.trim.2017.07.005 28778713

[33] Watanabe H Tsuchiya T Shimoyama K Shimizu A Akita S Yukawa H. Adipose-derived mesenchymal stem cells attenuate rejection in a rat lung transplantation model. J Surg Res. (2018) 227:17–27. doi: 10.1016/j.jss.2018.01.016 29804850

[34] Shi M Liu Z Wang Y Xu R Sun Y Zhang M. A pilot study of mesenchymal stem cell therapy for acute liver allograft rejection. Stem Cells Trans Med. (2017) 6:2053–61. doi: 10.1002/sctm.17-0134 PMC5702514 29178564

[35] Casiraghi F Perico N Podesta MA Todeschini M Zambelli M Colledan M. Third-party bone marrow-derived mesenchymal stromal cell infusion before liver transplantation: A randomized controlled trial. Am J Transplant. (2021) 21:2795–809. doi: 10.1111/ajt.16468 33370477

[36] Caplan HW Prabhakara KS Toledano Furman NE Zorofchian S Kumar A Martin C. Combination therapy with Treg and mesenchymal stromal cells enhances potency and attenuation of inflammation after traumatic brain injury compared to monotherapy. Stem Cells. (2021) 39:358–70. doi: 10.1002/stem.3320 PMC8634698 33368792

[37] Kauke-Navarro M Knoedler S Panayi AC Knoedler L Noel OF Pomahac B. Regulatory T cells: liquid and living precision medicine for the future of VCA. Transplantation. (2023) 107:86–97. doi: 10.1097/TP.0000000000004342 36210500

[38] Knoedler L Knoedler S Panayi AC Lee CAA Sadigh S Huelsboemer L. Cellular activation pathways and interaction networks in vascularized composite allotransplantation. Front Immunol. (2023) 14:1179355. doi: 10.3389/fimmu.2023.1179355 37266446 PMC10230044

[39] Chandran S Tang Q Sarwal M Laszik ZG Putnam AL Lee K. Polyclonal regulatory T cell therapy for control of inflammation in kidney transplants. Am J Transplant. (2017) 17:2945–54. doi: 10.1111/ajt.14415 PMC5662482 28675676

[40] Todo S Yamashita K Goto R Zaitsu M Nagatsu A Oura T. A pilot study of operational tolerance with a regulatory T-cell-based cell therapy in living donor liver transplantation. Hepatology. (2016) 64:632–43. doi: 10.1002/hep.28459 26773713

[41] Roemhild A Otto NM Moll G Abou-El-Enein M Kaiser D Bold G. Regulatory T cells for minimising immune suppression in kidney transplantation: phase I/IIa clinical trial. BMJ. (2020) 371:m3734. doi: 10.1136/bmj.m3734 33087345 PMC7576328

[42] Sawitzki B Harden PN Reinke P Moreau A Hutchinson JA Game DS. Regulatory cell therapy in kidney transplantation (The ONE Study): a harmonised design and analysis of seven non-randomised, single-arm, phase 1/2A trials. Lancet. (2020) 395:1627–39. doi: 10.1016/S0140-6736(20)30167-7 PMC7613154 32446407

[43] Hutchinson JA Riquelme P Brem-Exner BG Schulze M Matthai M Renders L. Transplant acceptance-inducing cells as an immune-conditioning therapy in renal transplantation. Transplant international: Off J Eur Soc Organ Transplant. (2008) 21:728–41. doi: 10.1111/j.1432-2277.2008.00680.x 18573142

[44] Schweizer R Waldner M Oksuz S Zhang W Komatsu C Plock JA. Adipose-derived stromal cell therapy combined with a short course non-myeloablative conditioning promotes long-term graft tolerance in vascularized composite allotransplantation. Am J Transplant. (2020) 20(5):1272–84. doi: 10.1111/ajt.15726 31774619

[45] Schweizer R Waldner M Oksuz S Zhang W Komatsu C Plock JA. Evaluation of porcine versus human mesenchymal stromal cells from three distinct donor locations for cytotherapy. Front Immunol. (2020) 11:826. doi: 10.3389/fimmu.2020.00826 32435248 PMC7218165

[46] Guo H Li B Li N Liu X Gao H Sun X. Exosomes: Potential executors of IL-35 gene-modified adipose-derived mesenchymal stem cells in inhibiting acute rejection after heart transplantation. Scand J Immunol. (2022) 96:e13171. doi: 10.1111/sji.13171 35398907

[47] Zhang W Lee PL Li J Komatsu C Wang Y Sun H. Local delivery of adipose stem cell promotes allograft survival in a rat hind limb model of vascularized composite allotransplantation. Plast Reconstr Surg. (2023). doi: 10.1097/PRS.0000000000010510 37014960

[48] Wu Y Zhao RC. The role of chemokines in mesenchymal stem cell homing to myocardium. Stem Cell Rev. (2012) 8:243–50. doi: 10.1007/s12015-011-9293-z 21706142

[49] Ren G Zhang L Zhao X Xu G Zhang Y Roberts AI. Mesenchymal stem cell-mediated immunosuppression occurs via concerted action of chemokines and nitric oxide. Cell Stem Cell. (2008) 2:141–50. doi: 10.1016/j.stem.2007.11.014 18371435

[50] Calligaris M Zito G Busa R Bulati M Iannolo G Gallo A. Proteomic analysis and functional validation reveal distinct therapeutic capabilities related to priming of mesenchymal stromal/stem cells with IFN-gamma and hypoxia: potential implications for their clinical use. Front Cell Dev Biol. (2024) 12:1385712. doi: 10.3389/fcell.2024.1385712 38882056 PMC11179434

[51] Soares MA Massie JP Rifkin WJ Rao N Duckworth AM Park C. Ex vivo allotransplantation engineering: Delivery of mesenchymal stem cells prolongs rejection-free allograft survival. Am J Transplant. (2018) 18:1657–67. doi: 10.1111/ajt.14668 29359512

[52] Cheng HY Anggelia MR Lin CH Lin CF. Preconditioned mesenchymal stromal cells to improve allotransplantation outcome. Cells. (2021) 10(9):2325. doi: 10.3390/cells10092325 34571974 PMC8469056

[53] Wang Y Wang S Gu C Xiong Y Shen H Liu F. Ex-vivo treatment of allografts using adipose-derived stem cells induced prolonged rejection-free survival in an allogenic hind-limb transplantation model. Ann Transl Med. (2020) 8:867. doi: 10.21037/atm-19-4730 32793711 PMC7396797

[54] Eggenhofer E Benseler V Kroemer A Popp FC Geissler EK Schlitt HJ. Mesenchymal stem cells are short-lived and do not migrate beyond the lungs after intravenous infusion. Front Immunol. (2012) 3:297. doi: 10.3389/fimmu.2012.00297 23056000 PMC3458305

[55] Kallmeyer K Andre-Levigne D Baquie M Krause KH Pepper MS Pittet-Cuenod B. Fate of systemically and locally administered adipose-derived mesenchymal stromal cells and their effect on wound healing. Stem Cells Trans Med. (2020) 9:131–44. doi: 10.1002/sctm.19-0091 PMC6954716 31613054

[56] Freitas GP Lopes HB Souza ATP Oliveira P Almeida ALG Souza LEB. Cell therapy: effect of locally injected mesenchymal stromal cells derived from bone marrow or adipose tissue on bone regeneration of rat calvarial defects. Sci Rep. (2019) 9:13476. doi: 10.1038/s41598-019-50067-6 31530883 PMC6748998

[57] Liu C Chen J Liu B Yuan S Shou D Wen L. Role of IL-18 in transplant biology. Eur Cytokine Netw. (2018) 29:48–51. doi: 10.1684/ecn.2018.0410 30078783

[58] Friedman O Carmel N Sela M Abu Jabal A Inbal A Ben Hamou M. Immunological and inflammatory mapping of vascularized composite allograft rejection processes in a rat model. PloS One. (2017) 12:e0181507. doi: 10.1371/journal.pone.0181507 28746417 PMC5528841

[59] Wolfram D Morandi EM Eberhart N Hautz T Hackl H Zelger B. Differentiation between acute skin rejection in allotransplantation and T-cell mediated skin inflammation based on gene expression analysis. BioMed Res Int. (2015) 2015:259160. doi: 10.1155/2015/259160 25756043 PMC4338383

[60] Messner F Etra JW Shores JT Thoburn CJ Hackl H Iglesias Lozano M. Noninvasive evaluation of intragraft immune responses in upper extremity transplantation. Transplant international: Off J Eur Soc Organ Transplant. (2021) 34:894–905. doi: 10.1111/tri.13854 33626223

[61] Kato T Okumi M Tanemura M Yazawa K Kakuta Y Yamanaka K. Adipose tissue-derived stem cells suppress acute cellular rejection by TSG-6 and CD44 interaction in rat kidney transplantation. Transplantation. (2014) 98:277–84. doi: 10.1097/tp.0000000000000230 24983309

[62] Roddy GW Oh JY Lee RH Bartosh TJ Ylostalo J Coble K. Action at a distance: systemically administered adult stem/progenitor cells (MSCs) reduce inflammatory damage to the cornea without engraftment and primarily by secretion of TNF-alpha stimulated gene/protein 6. Stem Cells. (2011) 29:1572–9. doi: 10.1002/stem.708 21837654

[63] Luzina IG Keegan AD Heller NM Rook GA Shea-Donohue T Atamas SP. Regulation of inflammation by interleukin-4: a review of “alternatives. J leukocyte Biol. (2012) 92:753–64. doi: 10.1189/jlb.0412214 PMC3441310 22782966

[64] Chen J Wang Y Hu H Xiong Y Wang S Yang J. Adipose-derived cellular therapies prolong graft survival in an allogenic hind limb transplantation model. Stem Cell Res Ther. (2021) 12:94. doi: 10.1186/s13287-021-02162-7 33514430 PMC7847016

[65] Jiang H Wynn C Pan F Ebbs A Erickson LM Kobayashi M. Tacrolimus and cyclosporine differ in their capacity to overcome ongoing allograft rejection as a result of their differential abilities to inhibit interleukin-10 production. Transplantation. (2002) 73:1808–17. doi: 10.1097/00007890-200206150-00019 12085006

[66] Devadoss CW Vijaya VM E M Venkataramana SR M SG. Tacrolimus associated localized thrombotic microangiopathy developing in early stage after renal transplantation. J Clin Diagn Res. (2012) 6:1786–8. doi: 10.7860/JCDR/2012/4535.2614 PMC3552231 23373055

[67] Halahleh K Arai Y Gavriilaki E. Post-transplant complications. Blood Cell Ther. (2023) 6:23–9. doi: 10.31547/bct-2022-021 PMC10266915 37324567

[68] Fenton M Simmonds J Shah V Brogan P Klein N Deanfield J. Inflammatory cytokines, endothelial function, and chronic allograft vasculopathy in children: an investigation of the donor and recipient vasculature after heart transplantation. Am J Transplant. (2016) 16:1559–68. doi: 10.1111/ajt.13643 26614396

[69] Sinha I Pomahac B. Split rejection in vascularized composite allotransplantation. Eplasty. (2013) 13:e53. 24244785 PMC3795428

[70] Przybylek B Boethig D Neumann A Borchert-Moerlins B Daemen K Keil J. Novel cytokine score and cardiac allograft vasculopathy. Am J Cardiol. (2019) 123:1114–9. doi: 10.1016/j.amjcard.2018.12.034 30660351

[71] Labarrere CA Woods JR Hardin JW Jaeger BR Zembala M Deng MC. Early inflammatory markers are independent predictors of cardiac allograft vasculopathy in heart-transplant recipients. PloS One. (2014) 9:e113260. doi: 10.1371/journal.pone.0113260 25490200 PMC4260824

[72] Bai X Qi Z Song G Zhao X Zhao H Meng X. Effects of monocyte chemotactic protein-1 and nuclear factor of kappa B pathway in rejection of cardiac allograft in rat. Transplant Proc. (2015) 47:2010–6. doi: 10.1016/j.transproceed.2015.05.014 26293090

[73] Saiura A Sata M Hiasa K Kitamoto S Washida M Egashira K. Antimonocyte chemoattractant protein-1 gene therapy attenuates graft vasculopathy. Arterioscler Thromb Vasc Biol. (2004) 24:1886–90. doi: 10.1161/01.ATV.0000141045.49616.6f 15284091

[74] Zhao H Zhang Y Song G Zhao W Bai X Zhang J. The significance for chronic rejection of cardiac allograft of regulated upon activation normal T-cell cytokine and its CCR5 receptor. Transplant Proc. (2013) 45:635–8. doi: 10.1016/j.transproceed.2012.03.065 23498802

[75] van Loosdregt J van Oosterhout MF Bruggink AH van Wichen DF van Kuik J de Koning E. The chemokine and chemokine receptor profile of infiltrating cells in the wall of arteries with cardiac allograft vasculopathy is indicative of a memory T-helper 1 response. Circulation. (2006) 114:1599–607. doi: 10.1161/CIRCULATIONAHA.105.597526 17015796

[76] Liu L Fang C Fu W Jiang B Li G Qin L. Endothelial cell-derived interleukin-18 released during ischemia reperfusion injury selectively expands T peripheral helper cells to promote alloantibody production. Circulation. (2020) 141:464–78. doi: 10.1161/CIRCULATIONAHA.119.042501 PMC7035199 31744330

[77] Pober JS Chih S Kobashigawa J Madsen JC Tellides G. Cardiac allograft vasculopathy: current review and future research directions. Cardiovasc Res. (2021) 117:2624–38. doi: 10.1093/cvr/cvab259 PMC8783389 34343276

[78] Lin-Wang HT Cipullo R Dias Franca JI Finger MA Rossi Neto JM Correia EB. Intragraft vasculitis and gene expression analysis: Association with acute rejection and prediction of mortality in long-term heart transplantation. Clin Transplant. (2018) 32:e13373. doi: 10.1111/ctr.13373 30080295

