# Supplementary material for: Donor adipose-derived stromal cells are vasoprotectant but unable to revert acute rejection in rodent vascularized composite allotransplants
Source: Front Immunol. 2025 Apr 28;16:1581599. doi: 10.3389/fimmu.2025.1581599 (PMC12066311; doi:10.3389/fimmu.2025.1581599)
Supplement: Supplementary file 1 [file DataSheet1.pdf]

Supplementary Figures

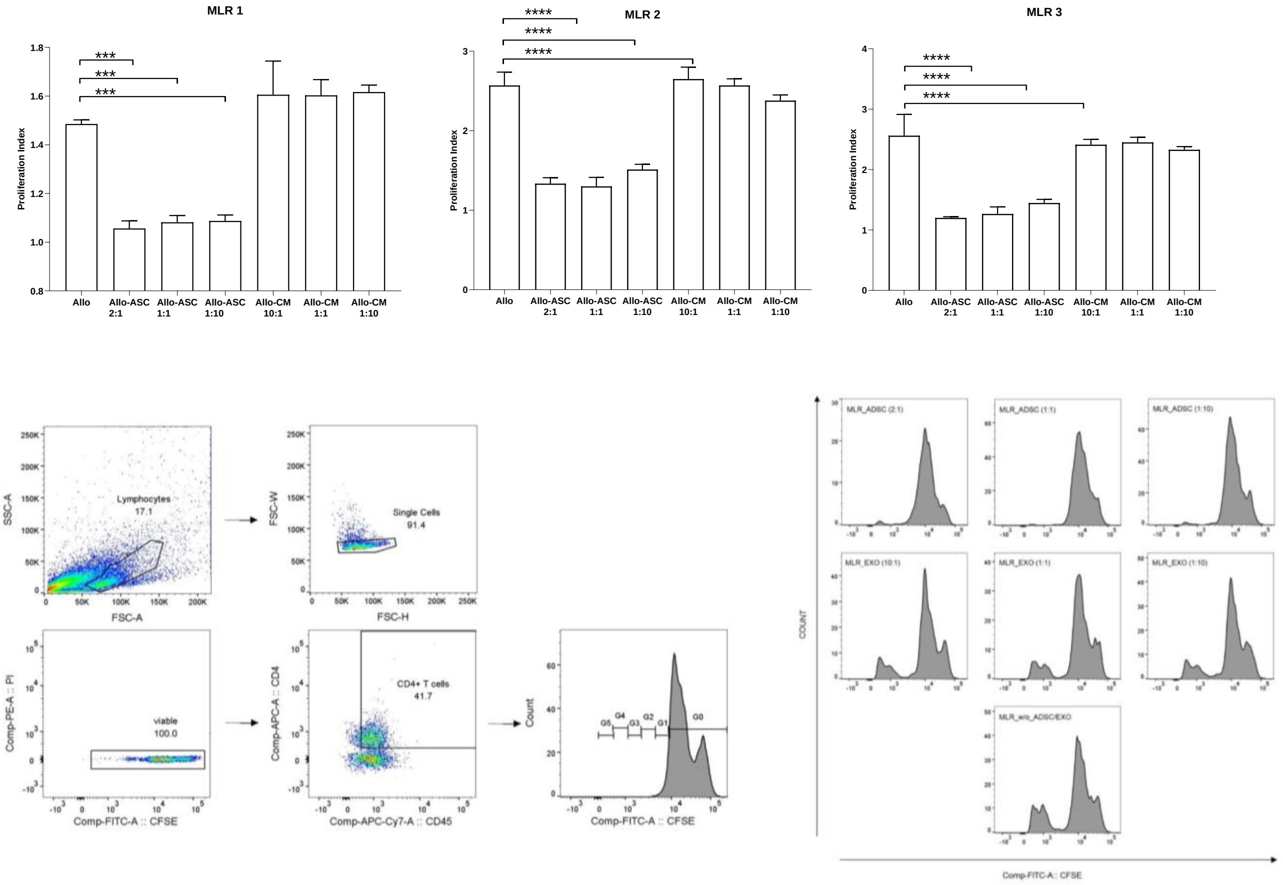

Figure 1. Three distinct mixed lymphocyte reaction assays are shown in this figure's above panel. An exemplary workup is shown in the panel below, for investigation of proliferation of responder cells using CFSE staining, assessed on $CD4^+CD45^+$ T cells by flow cytometry.

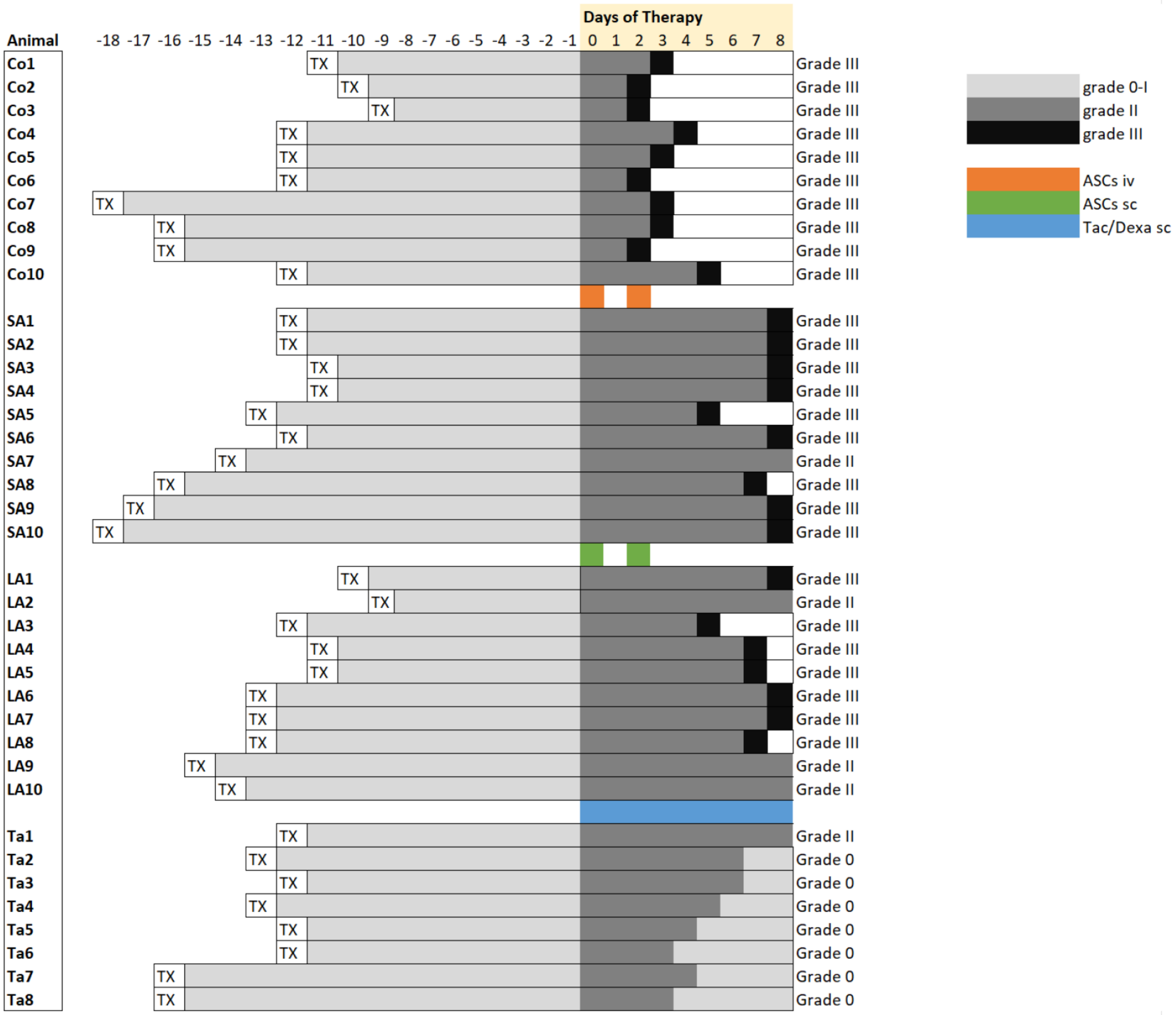

Figure 2. Specific follow up and outcome after transplantation for each animal of the different groups. Days from transplantation (Tx) until first therapy are illustrated. The different gray shades represent different rejection grades, while the different colors represent different therapy modalities as shown in the legend. ASC = Adipose-derived Stem Cell; Co = controls; LA = local ASC group; SA = systemic ASC group; Ta = FK-506 group. Tx = transplantation.

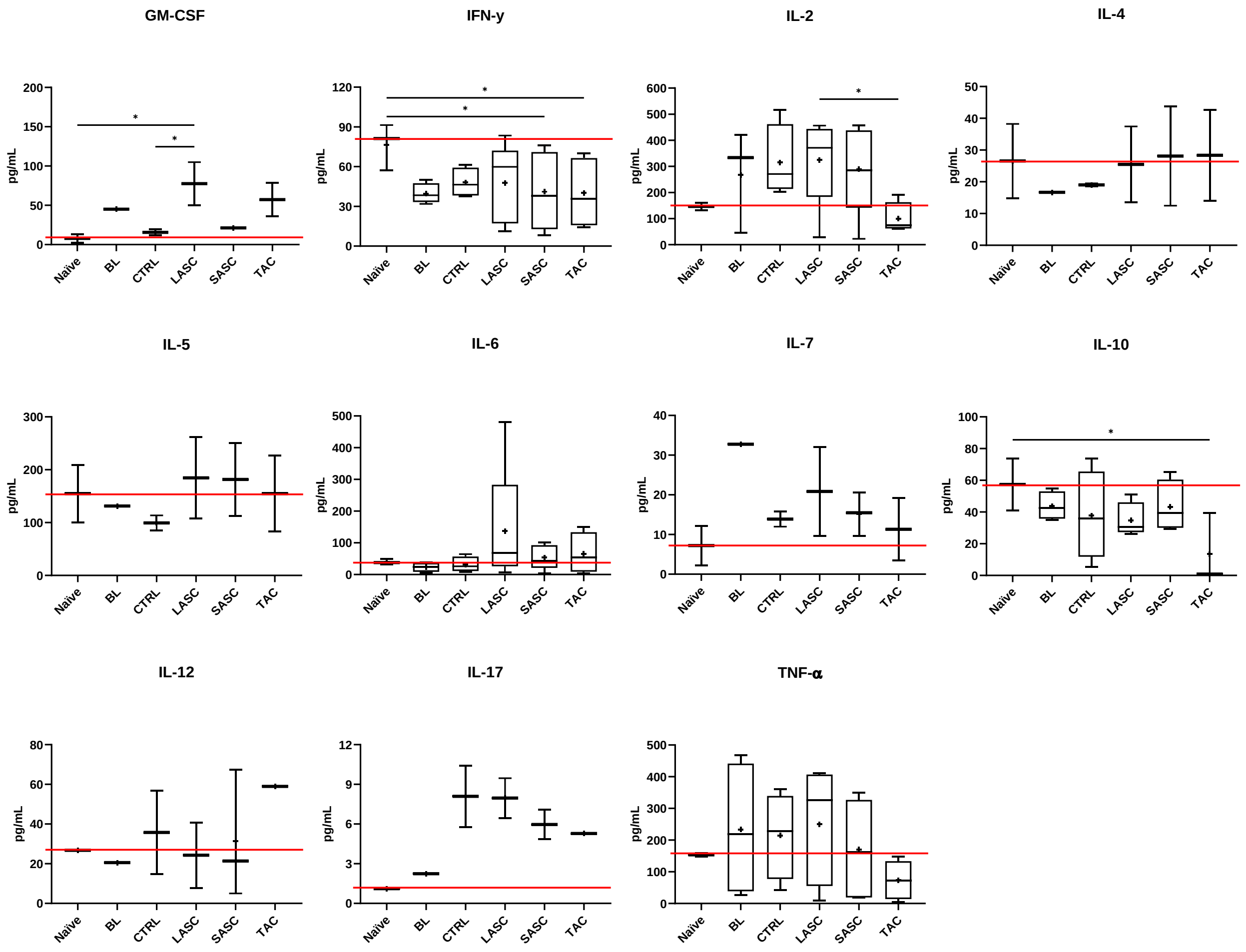

Figure 3. Immune cytokine values in animal's blood samples not included in the main manuscript are depicted here. BL = baseline = at grade II rejection, prior to therapy (cell therapy or FK-506 therapy). Red line = naïve mean value threshold. ASC = Adipose-derived Stem Cell; CTRL = controls; LASC = local ASC group; SASC = systemic ASC group; TAC = FK-506 group.

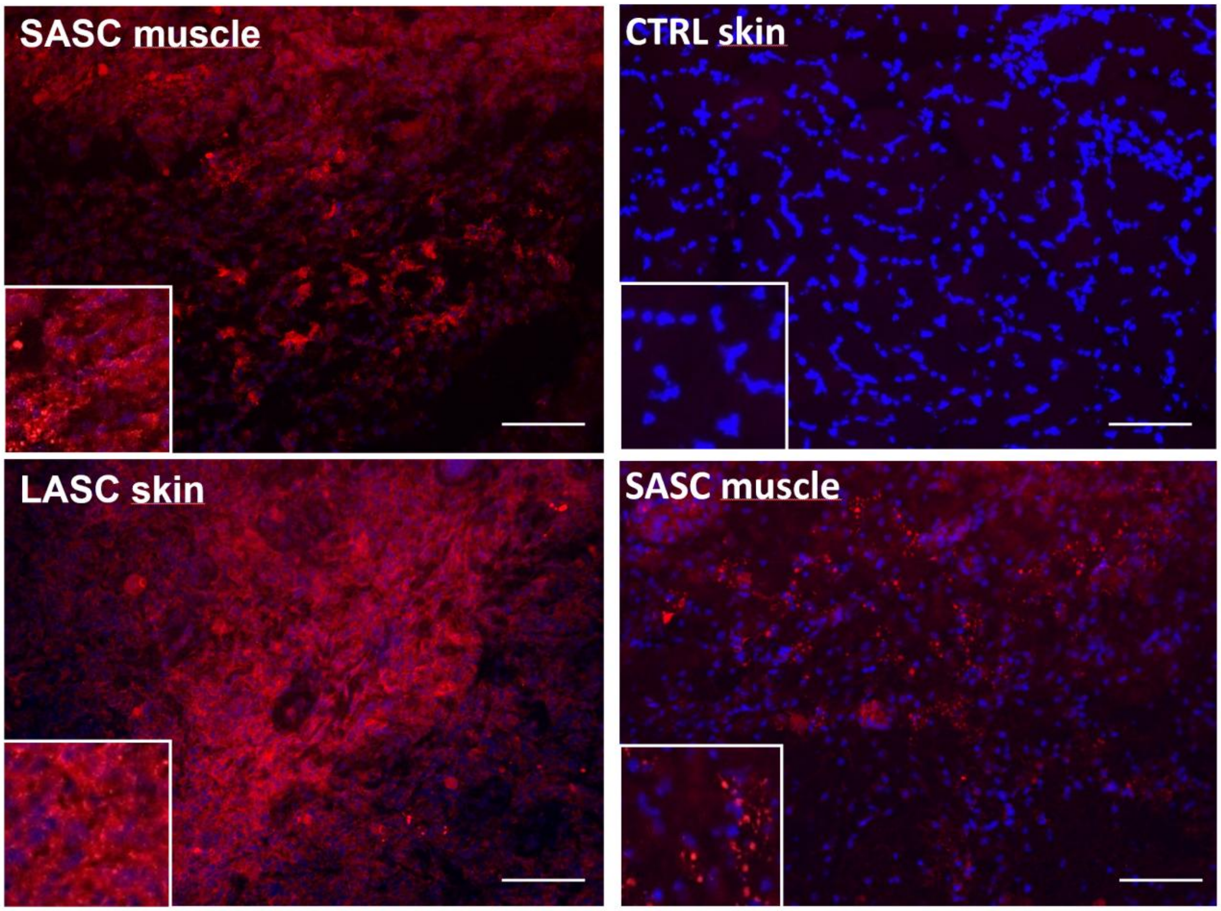

Figure 4. Homing and localization of injected ASCs. At endpoint PKH-26-labelled ASCs were identified (red signal). Blue DAPI stain. Pictures taken at 10x magnification, bar = 50 µm. ASC = Adipose-derived Stem Cell; CTRL = controls; LASC = local ASC group; SASC = systemic ASC group.

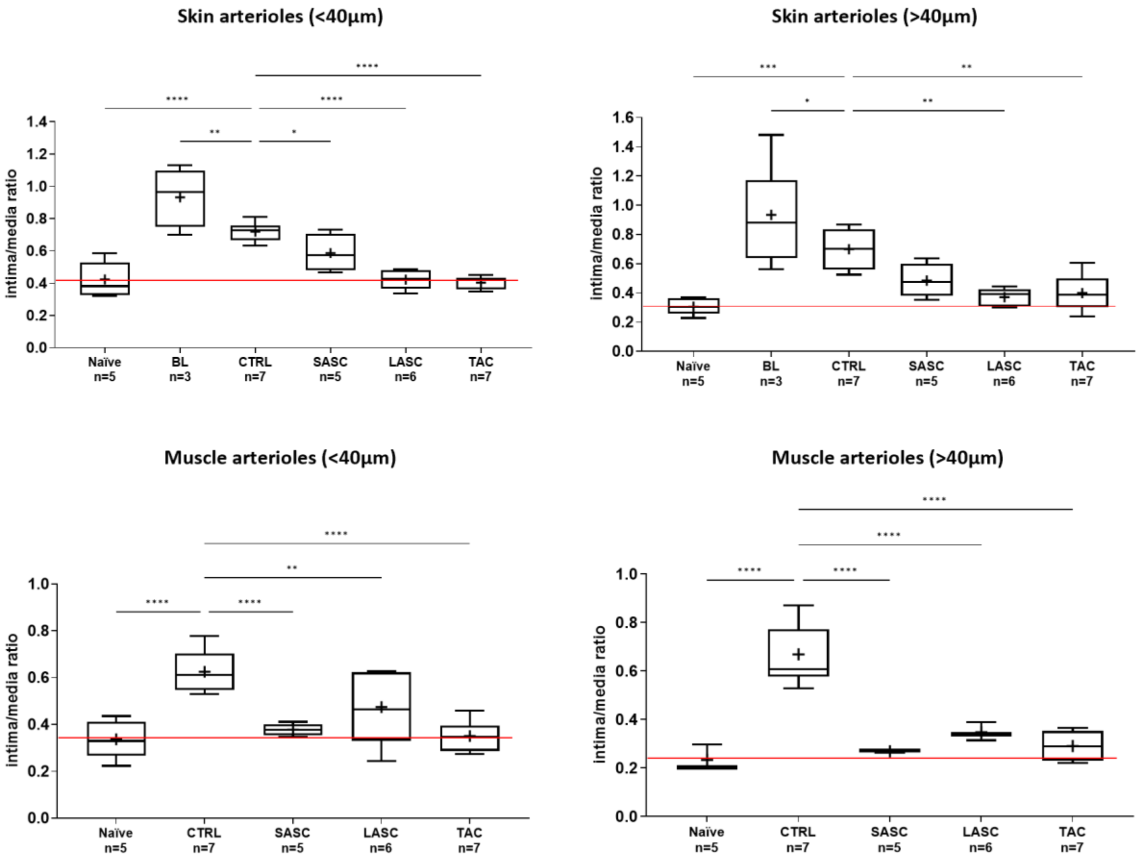

Figure 5. The intima/media ratio was measured in allograft arterioles of skin and muscle tissue. The arterioles are divided in large (right) and small (left) arterioles (cutoff 40µm). An additional sample (BL = baseline) was taken for skin assessment prior to start of cell or FK-506 therapy. ASC = Adipose-derived Stem Cell; CTRL = controls; LASC = local ASC group; SASC = systemic ASC group; TAC = FK-506 group. Red line = naïve mean value threshold.

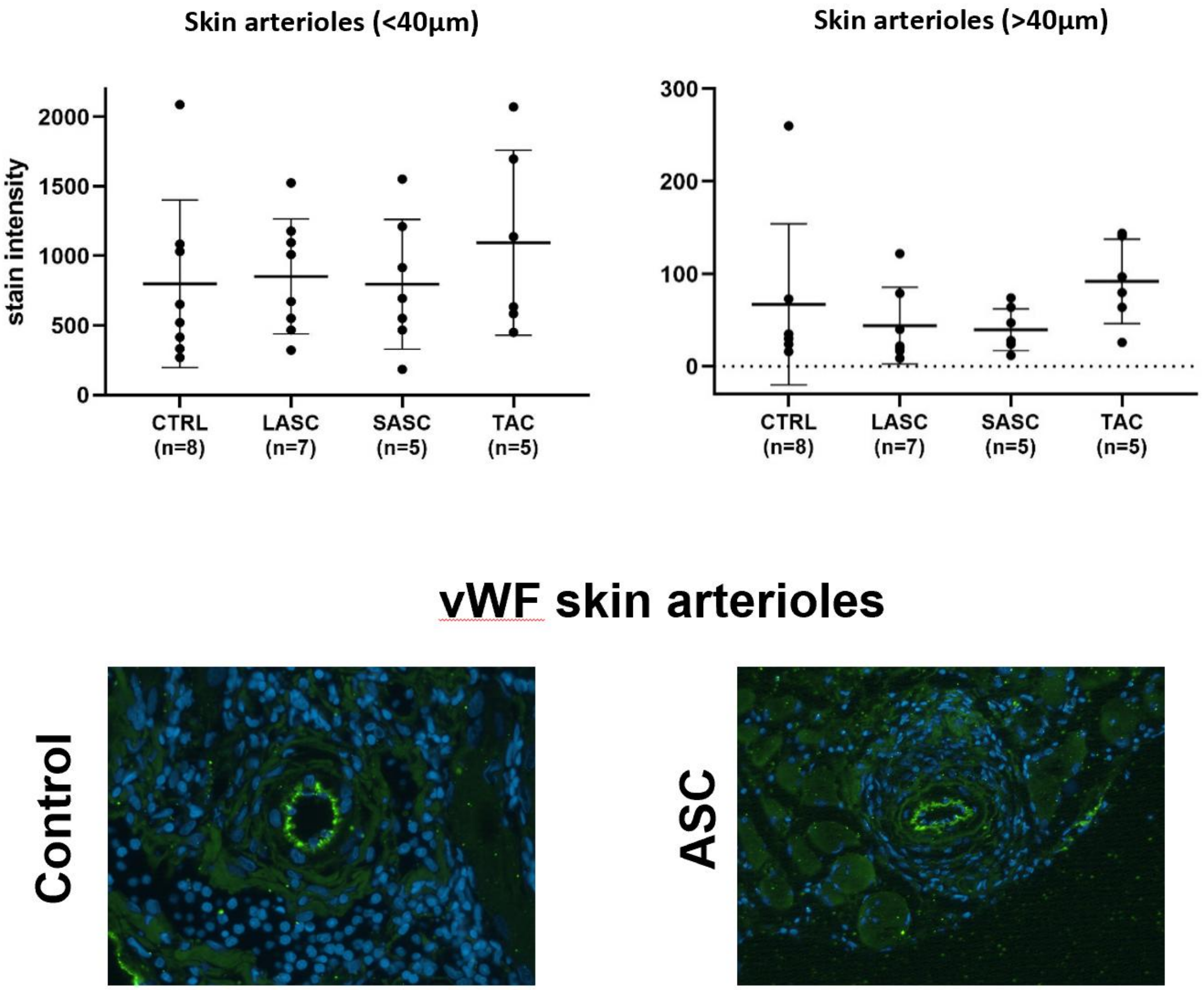

Figure 6. vWF expression in large and small skin arterioles is depicted in the above graphs for the different groups. Two exemplary fluorescence microscopy images are provided for a control and cell therapy animals. ASC = Adipose-derived Stem Cell; CTRL = controls; LASC = local ASC group; SASC = systemic ASC group; TAC = FK-506 group; vWF = von Willebrand factor

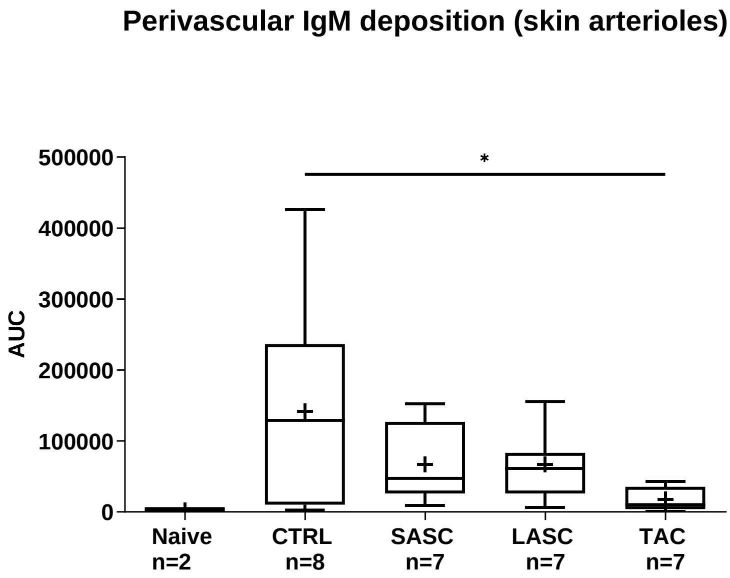

Figure 7. Perivascular IgM deposition around skin arterioles in the different groups is depicted in this graph. ASC = Adipose-derived Stem Cell; AUC = Area under the curve; CTRL = controls; LASC = local ASC group; SASC = systemic ASC group; TAC = FK-506 group. * $p<0.05$.
